# A data-driven model of the COVID-19 spread among interconnected populations: epidemiological and mobility aspects following the lockdown in Italy

**DOI:** 10.1007/s11071-021-06840-2

**Published:** 2021-09-03

**Authors:** Paolo Di Giamberardino, Daniela Iacoviello, Federico Papa, Carmela Sinisgalli

**Affiliations:** 1 Department of Computer, Control and Management Engineering A. Ruberti, Sapienza University of Rome, Rome, Italy; 2 Institute for Systems Analysis and Computer Science “A. Ruberti” - CNR, Rome, Italy

**Keywords:** COVID-19, Multi-group epidemic ODE model, COVID-19 spread in Italy, System control and identification

## Abstract

An epidemic multi-group model formed by interconnected SEIR-like structures is formulated and used for data fitting to gain insight into the COVID-19 dynamics and into the role of non-pharmaceutical control actions implemented to limit the infection spread since its outbreak in Italy. The single submodels provide a rather accurate description of the COVID-19 evolution in each subpopulation by an extended SEIR model including the class of asymptomatic infectives, which is recognized as a determinant for disease diffusion. The multi-group structure is specifically designed to investigate the effects of the inter-regional mobility restored at the end of the first strong lockdown in Italy (June 3, 2020). In its time-invariant version, the model is shown to enjoy some analytical stability properties which provide significant insights on the efficacy of the implemented control measurements. In order to highlight the impact of human mobility on the disease evolution in Italy between the first and second wave onset, the model is applied to fit real epidemiological data of three geographical macro-areas in the period March–October 2020, including the mass departure for summer holidays. The simulation results are in good agreement with the data, so that the model can represent a useful tool for predicting the effects of the combination of containment measures in triggering future pandemic scenarios. Particularly, the simulation shows that, although the unrestricted mobility alone appears to be insufficient to trigger the second wave, the human transfers were crucial to make uniform the spatial distribution of the infection throughout the country and, combined with the restart of the production, trade, and education activities, determined a time advance of the contagion increase since September 2020.

## Introduction

The “coronavirus disease 2019” (COVID-19), caused by SARS-CoV-2, posed novel challenges to all world countries, often evidencing their vulnerability in the practical management of emergency states, particularly concerning the health effects, but also the implications for economic growth and social development. Understanding and quantifying the dominant variables that govern the current pandemic evolution, especially in order to limit the impact of future outbreaks, imposes the need of framing the determinants of the epidemic dynamics [48]. Many literature studies take into account several types of possible interventions, mainly identifying two domains of variables: pathogen-associated variables and society-based variables. This latter variable domain appears to be particularly relevant for COVID-19, since individuals are actually the vectors of SARS-CoV-2 virus.

Thus, although vaccines are currently available, social distancing and personal protective measures are still important mechanisms for controlling the COVID-19 spread. The effectiveness of social distancing is studied by many authors, whose papers propose projections where the impact of containment measures in reducing the infection spread is shown [8, 11, 16, 27, 44].

In the early stage of pandemic, only qualitative data analysis was performed, as in [30] where data regarding how the human mobility changed in the USA at the beginning of the pandemic course are studied; in that paper, it is also stressed the importance of quantifying the social distancing practices, emphasizing the opportunity of determining relationships between confirmed cases and the social distancing plateau. The concept of “social distancing” can also be associated with travel restrictions, that is to the attempt of limiting the virus transmission by reducing the amount of travels among regions. One of the first studies about the effects of human mobility on COVID-19 spread is proposed in [28]. Through the analysis of time mobility data from Wuhan, the study shows the importance of applying travel restrictions in the early stage of the outbreak, evidencing their lack of effectiveness in late stages.

Among the growing number of literature papers on the topic, we focus on the quantitative studies concerning the effects of limiting social distancing and human mobility. These studies are generally based on mathematical modelling, still exploiting rather different approaches, and most often they use public or volunteered datasets to assess the impact of different non-pharmaceutical countermeasures, [12, 13, 23, 39, 40]. As an example, we mention the works of correlational analysis based on (generalized) regression models of city clusters; in particular, applications are mainly proposed for single countries, e.g., China [53], USA countries [2], and some European countries (namely France, Spain, and Italy) [25], or for cross-city analysis in many worldwide countries [47]. In [7], a framework that employs an epidemic Renormalization Group (eRG) approach to model the effects of inter- and extra-European border control and of social distancing for single countries is proposed; the model describes the time-evolution of the infected cases in a specific isolated region, while including the interactions among multiple regions of the World.

In many recent works, both deterministic/stochastic and discrete/continuous models have been applied for the description, forecast and control of the COVID-19 epidemic spread. In the framework of deterministic compartmental models, the classes of Susceptible (S), Exposed (E), Infected (I), and Removed (R) subjects are generally introduced, yielding SEIR models. For the COVID-19 pandemic, because of the specificity of the disease, other categories are generally introduced referring to the condition of infected patients.

In this respect, notable modeling setups take into account the symptomatology level, thus distinguishing presymptomatic and asymptomatic infected individuals [41], with the addition of the quarantined class [16, 17] or acutely and mildly infection level along with the conditions of hospitalization and home-isolation [21, 24].

Different modeling approaches can include distributed time delays[50] or the computational model using probabilistic cellular automata [22, 32].

Moreover, among the most recent (and actually huge) literature on the COVID-19 modeling, we also mention some papers proposing interesting variants of the classical SIR model and using different approaches: the age-structured SIR model [36], the stochastic, discrete, age-structured compartmental model [1] in which distributed time delays related to the periods of incubation, infection, and quarantine are considered as in [35]. Another aspect common to these latter works that matters in relation to our study is that they analyze the first epidemic period (until October 2020) in order to highlight how different strategies implemented in that peculiar time interval can affect the future epidemic trajectory.

It is important to stress that a particularly critical epidemiological characteristic has been recognized in the fraction of infectious cases remaining undocumented owing to mild or very limited symptoms; indeed, estimating the extent of undiagnosed infections is crucial for evaluating the overall prevalence and contagiousness and then the pandemic potential of the disease, [17, 31].

Some variants of the original SIR/SEIR framework, modeling peculiar aspects of epidemic transmission dynamics, have been proposed in the past to generate insights into the evolution of specific infectious diseases and assess the potential impact of different intervention strategies; so, these studies can usefully support the research on the present COVID-19 pandemic. Among numerous modeling works, we mention the book [9] and the paper [46], presenting in-depth overviews of theoretical and applicative results on measles, ebola, and other viral infections. Examples of more specific works are [15, 43] on the measles disease, and [14, 37, 38] on HIV/AIDS. Also, it is worth mentioning that very recent works deal with the impact of the COVID-19 co-infection in patient with pre-existent morbidity [11]; for example, [4] reports a statistical population-based study to estimate risk and possible outcome of the association between HIV infection and COVID-19.

The epidemic models of the kind described concern the transmission of the virus *within* population. Concerning aspects of the virus transmission *between* populations, multi-group epidemic models, which are suitable extensions of SIR/SEIR frameworks, can be used to represent the COVID-19 spread among different (heterogeneous) populations, and to study the effect of interactions and travel restrictions on the pandemic evolution, [3, 6, 10, 20, 33, 34, 42]. The heterogeneity of the subpopulations is intended with respect to the epidemic properties and can depend on their different geographical allocation or other structural variables (e.g., economy, age, mobility).

The model presented in this paper is based on a previous epidemic model reported in [17], which represented the COVID-19 evolution by a SEIR-type model including two subpopulations of infected subjects, undiagnosed and diagnosed, and explicitly accounting for a fraction of asymptomatic infective subjects. The present model structure, depicted in Sect. 2, incorporates *N* interconnected epidemic models of that kind, particularly with the aim of representing the effects of individual interactions and geographical exchanges among groups. In Sect. 3, analytical results on the dynamics of both the isolated subsystem and the interconnected model are given. In Sect. 4, the general model is specialized for $N=3$ and it is applied to simulate the disease evolution in Italy, and precisely in three macroareas, in order to evidence some interesting aspects related to the increased human mobility following the first pandemic wave. The numerical results show that the model is apt in describing the summer period 2020 of the COVID-19 epidemic in Italy and the effect of the holiday exodus from North to Center-South, by explicitly accounting for different scenarios characterizing geographically distinct macro-areas of the Italian territory (northern, central, and southern). The detailed analysis reported in Sect. 4 includes two periods: (i) the first one characterized by the strong lockdown implemented in our country and (ii) the following reopening period going from the control relaxation (beginning of June 2020) until new social, economic and mobility restrictions were implemented to contain the second contagion wave (mid-October 2020). As explained in the following, the time period selected for the analysis, as well as the number and the geographical localization of the sub-groups considered, are actually motivated by the main goal of this paper, that is the in-depth analysis of the human mobility (satisfactorily captured in this period by the assumed model structure) after the removal of the containment measures following the first severe lockdown. However, in Sect. 5, the same model structure is exploited to evaluate the mobility impact after the period of interest, extending the analysis to the present days and confirming some epidemiological and mobility aspects already highlighted in Sect. 4.

## A multi-group epidemic model for the spread of COVID-19 among *N* groups

The model proposed here has a multi-group structure that incorporates different subunits, each one describing the dynamic evolution of the COVID-19 within a homogeneous population, whose epidemic evolution differs from that of all other units. For instance, different groups can represent different geographical areas or structurally different populations. The *N* groups (namely subunits) are interconnected by a mobility network that accounts for the transfers of individuals who are allowed to travel from a group to another. Most typically, the model can describe a geographical system composed by *N* regions with people of each region moving for work, study or simply personal/holiday reasons.

**Fig. 1:**
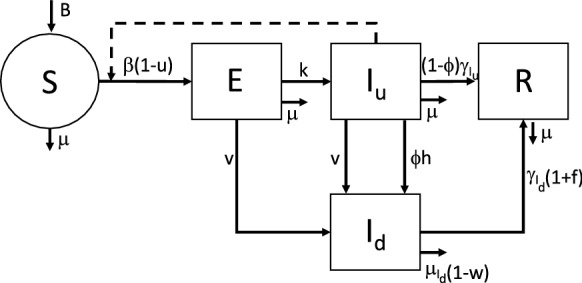
Block diagram of the epidemic model representing a single subpopulation

Each of the *N* submodels is a simplified version of the model previously proposed in [17] for the description of the first phase of the epidemic spread in our country (thereby modeled as a whole homogeneously mixing group). In particular, the compartment of subjects isolated while waiting for the results of the swab tests, included in [17], has been removed due to the increasing capability of doing fast tests along the considered period, especially at the end of lockdown, which provide almost immediate responses of positiveness (so producing a new diagnosed and isolated infected) or negativeness (leaving the tested subject in the class of susceptible people). In addition to a compartment of exposed individuals, which is proper of SEIR models, our model explicitly distinguishes between diagnosed and undiagnosed infective patients. As shown in [17], the proposed structure appears appropriate to mimic the Italian case, by incorporating also control actions reproducing government restrictions and emergency actions implemented to detect the infected cases, especially asymptomatic or mildly symptomatic ones. The block diagram reported in Fig. 1 shows the general structure and the state variables of one regional submodel.

Precisely, each submodel takes into account the following five state variables:

$$S_i(t)$$,
number of susceptible individuals;
$$E_i(t)$$,
number of exposed (infected but not yet infective) individuals;
$$I_{u_i}(t)$$,
number of undiagnosed infective patients, accounting for two subpopulations: (i) asymptomatic or developing mild or limited symptoms during their whole infection period; (ii) developing, at a certain point, recognizable symptoms, still remaining undocumented;
$$I_{d_i}(t)$$,
number of diagnosed infective patients, receiving medical treatments (to cure the infection or its complications). It is assumed that they cannot transmit the virus since they are isolated (at home or at hospital);
$$R_i(t)$$,
number of healed patients (spontaneously or after therapy).

**Fig. 2:**
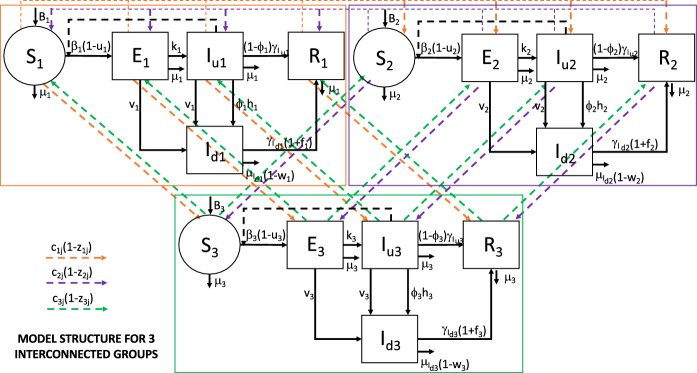
Block diagram of a mobility scheme among $N=3$ interconnected groups of epidemic diffusion. Controls for group *i*: $u_i \in [0,1]$, social contact limitations; $v_i \in [0,1]$, test campaign intensity; $w_i \in [0,1]$, efficacy of therapies against COVID-19 complications; $f_i \ge 0$, efficacy of therapies against COVID-19; $z_{i,j} \in [0,1]$, limitation of movements between groups *i* and *j*. Coefficients $c_{i,j}$: transition probability of a subject from compartment *i* to compartment *j*

The complete model formulation includes *N* structurally identical groups or subsystems, with each group *i*, $\{i = 1, 2, \ldots , N\}$, described by a SEIR-type model with undiagnosed and diagnosed infected subjects, as reported above. For sake of compactness, when the whole system is considered, a vectorial notation is introduced for the state space variables, defining the vector

1 $$\begin{aligned} S(t)=\begin{pmatrix} S_1(t)&\cdots&S_N(t) \end{pmatrix}^T \end{aligned}$$

and, with the same procedure, *E*(*t*), $I_u(t)$, $I_d(t)$ and *R*(*t*). The *N* subsystems are connected by means of a mobility network allowing people to move among groups. In the following, we refer to a specific epidemic group by its identifier *i*, also using the same subscript to denote the related state variables and parameters. In general, however, we assume that the only individuals allowed to move are the ones having no evidence and/or diagnosis of infection, i.e., the ones belonging to compartments $S_i$, $E_i$, $I_{u_i}$, $R_i$, $i = 1, 2, \ldots , N$. Fig. 2 shows how the mobility network works for $N = 3$ subsystems, also illustrating the epidemic core model of each subsystem.

**Fig. 3:**
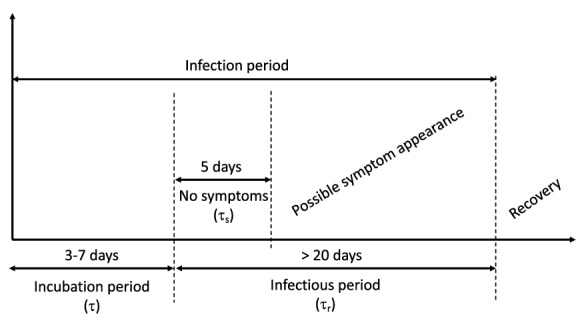
Schematic picture of the disease progression

So, the spread of COVID-19 among *N* epidemiologically distinct groups can be formally described by means of *N* systems of time-varying ODE models of the following kind:

2 $$\begin{aligned} \dot{S_i}= & {} B_i-\beta _i(1-u_i)S_i I_{u_i}-\mu _iS_i \nonumber \\&-\sum _{j=1, {j \ne i}}^{N}c_{i,j}(1-z_{i,j})S_i+\sum _{j=1, {j \ne i}}^{N}c_{j,i}(1-z_{j,i})S_j, \end{aligned}$$

3 $$\begin{aligned} \dot{E_i}= & {} \beta _i(1-u_i)S_iI_{u_i}-v_iE_i-k_iE_i-\mu _i E_i\nonumber \\&-\sum _{j=1, {j \ne i}}^{N}c_{i,j}(1-z_{i,j})E_i+\sum _{j=1, {j \ne i}}^{N}c_{j,i}(1-z_{j,i})E_j+{\varLambda }_{E_i},\nonumber \\ \end{aligned}$$

4 $$\begin{aligned} {\dot{I}}_{u_i}= & {} k_iE_i-v_iI_{u_i}-h_i\phi _iI_{u_i}-\gamma _{I_{u_i}}(1-\phi _i)I_{u_i}-\mu _i I_{u_i} \nonumber \\&-\sum _{j=1, {j \ne i}}^{N}c_{i,j}(1-z_{i,j})I_{u_i}+\sum _{j=1, {j \ne i}}^{N}c_{j,i}(1-z_{j,i})I_{u_j}+{\varLambda }_{I_{u_i}},\nonumber \\ \end{aligned}$$

5 $$\begin{aligned} {\dot{I}}_{d_i}= & {} h_i\phi _iI_{u_i}+v_i (E_i+I_{u_i})-\gamma _{I_{d_i}}(1+f_i)I_{d_i} \nonumber \\&-\mu _{I_{d_i}}(1-w_i)I_{d_i}, \end{aligned}$$

6 $$\begin{aligned} \dot{R_i}= & {} \gamma _{I_{u_i}}(1-\phi _i) I_{u_i}+\gamma _{I_{d_i}}(1+f_i)I_{d_i}-\mu _i R_i \nonumber \\&-\sum _{j=1, {j \ne i}}^{N}c_{i,j}(1-z_{i,j})R_i+\sum _{j=1, {j \ne i}}^{N}c_{j,i}(1-z_{j,i})R_j, \end{aligned}$$

where $i = 1,2, \ldots , N$. Now, we briefly explain the meaning of all the quantities included in the ODE system (2)-(6). $B_i$ is the net input rate in compartment $S_i$, which accounts for both the newborn (susceptible) individuals and the balance between immigration and emigration; $\mu _i$ is the per capita death rate owing to causes not related to the infection (natural death of the population) and it represents the loss rate from any compartment of the model except for $I_{d_i}$; $\mu _{I_{d_i}}$ is the per capita death rate of diagnosed patients $I_{d_i}$; $\beta _i$ is the relative contagiousness of individuals in compartment $I_{u_i}$ and it accounts for two main factors, which are the contagion probability from one infected-susceptible contact (related to the aggressiveness of the virus) and the frequency of contacts; $\phi _i$ represents the fraction of the infective population $I_{u_i}$ that will show recognizable symptoms and that will consequently be diagnosed and isolated (possibly receiving therapies); $k_i$ describes the transition from $E_i$ to $I_{u_i}$, and it is set to $k_i = 1/\tau _{i}$, where $\tau _{i}$ is the mean length of the incubation period (see Fig. 3); $h_i$ refers to the transition from $I_{u_i}$ to $I_{d_i}$, taken as $h_i = 1/\tau _{s_i}$, where $\tau _{s_i}$ is the average time from infection until the occurrence of the first recognizable symptoms; $\gamma _{I_{u_i}}$ models the outflow from the infective compartment $I_{u_i}$ associated to recovery from infection and, then, it is assumed $\gamma _{I_{u_i}}= 1/\tau _{r_i}$, with $\tau _{r_i}$ the mean recovery period without any medical assistance; similarly $\gamma _{I_{d_i}}$ models the outflow from the infective compartments $I_{d_i}$ due to recovery from the infection and, then, it is $\gamma _{I_{d_i}}= 1/{\tilde{\tau }}_{r_i}$, with ${\tilde{\tau }}_{r_i}$ denoting the mean recovery period of monitored patients; $c_{ij}$ is a weight accounting for the transition probability of a subject moving from the *i*-th compartment $S_i$ or $E_i$ or $I_{u_i}$ to the corresponding *j*-th compartment. Note that the coefficients $c_{ij}$ can also be time-varying in order to represent the variation of transfer probabilities possibly occurring over time. This variability is especially required for long-term analysis when “ordinary” mobility regimens alternate with highly “intense” transfer periods, like summer or Christmas or Easter seasons. Note also that, for the sake of generality, the recovery rates from compartments $I_{u_i}$ and $I_{d_i}$ are taken into account by the separate rate constants $\gamma _{I_{u_i}}$ and $\gamma _{I_{d_i}}$ respectively, even though to a first approximation, and in the absence of experimental evidences, they are assumed equal in the following simulation.

As far as the control actions are concerned, the time-varying quantities $u_i(t)$, $v_i(t)$, $w_i(t)$, $z_{i,j}(t)$, taking values in [0, 1], and $f_i(t)\ge 0$ are introduced to represent the intervention measures adopted by the authorities to contain the disease outbreak. More precisely, $u_i(t)$ quantifies possible actions locally implemented by authorities to reduce the contact rate, and then the relative infectivity $\beta _i$, of population *i*. It accounts for all the government decrees introduced to limit the physical interactions among people, but also for the informative campaign about hygienic measures, TV/radio announcements, and so on. The quantity $v_i(t)$ represents the intensity of the swab test campaign performed on subpopulation *i*, which changes daily depending on the number of swab tests actually performed. For the sake of simplicity, and in the absence of other indications, we assume that the amount of performed tests is equally distributed among people of compartments $S_i$, $E_i$ and $I_{u_i}$, so that the same per capita test rate can be assumed for all these compartments. This implies that the exit fluxes of tested (positive) individuals leaving $E_i$ and $I_{u_i}$ are proportional to the number of individuals within the same compartment, i.e., $v_i(t) E_i(t)$ and $v_i (t)I_{u_i}(t)$, respectively. We notice that the flux $v_i(t) S_i(t)$ of (negative) test results exiting $S_i$ does not explicitly appear in the model equation (see also Fig. 1) since it does not contribute to the dynamical evolution (actually an identical flux amount comes back to compartment $S_i$). The control actions $w_i(t)$ and $f_i(t)$ refer to the efficacy of the therapies adopted by the *i*th health system, either to reduce side effects of COVID-19 and, respectively, to cure the infection. Furthermore, the time-varying controls $z_{i,j}(t)$, $i,j = 1, 2, \ldots , N$, represent the interventions and mobility restrictions implemented by the central government or local authorities to limit people transfers between groups *i* and *j*.

Finally, the pair of input fluxes ${\varLambda }_{E_i}(t)$ and ${\varLambda }_{I_{u_i}}(t)$ is introduced (see Eqs. (3), (4)) to model the cumulative entry of infected people coming from outer groups/areas whose epidemic dynamics is not incorporated in the *N* group model. Since documented infective people are not allowed to travel, it is reasonable to account for such cumulative outer inputs only in the equations of $E_i$ and $I_{u_i}$, $i = 1, \ldots , N$.

We notice that the proposed model does not incorporate the possibility of re-infection. Indeed in our model, once recovered, a patient (R) is no longer susceptible of infection and cannot re-enter the S class. This simplifying hypothesis, which is actually the object of clinical studies and debates on the persistence and actual length of the immunity period, can be a valid assumption if a short-term analysis, like the one presented in the next section, is performed.

As a remark on the asymptomatic undiagnosed subjects, we observe that a susceptible subject (of any group *i*) can become infected if a non-safe contact with an infected undiagnosed subject occurs. During the infectious period that follows incubation, the newly infected subject can at some time develop recognizable symptoms being easily diagnosed, and possibly recovering after medication and assistance without serious consequences. However, in a number of cases, the infectious individual can be asymptomatic or mildly symptomatic until full recovery, remaining hidden and undocumented as a positive case in $I_{u_i}$. So, in fact, the class of asymptomatic undiagnosed subjects represents the most dangerous class responsible for the possible epidemic spread since the individuals are allowed to move, thus transmitting the contagion and increasing the number of infections also to other groups. Exhaustive swab test campaigns performed on the entire population can improve the capability of diagnosis contributing to mitigate the infection diffusion as reported in [29].

## Equilibria and stability analysis

The first step of the analysis, in Subsection 3.1, takes into account each *i*th subsystem as an isolated dynamical model, neglecting all the mobility contributions, i.e., setting $z_{i,j}=1$, $i,j=1,\dots ,N$. In order to take into account the actual situation in which control actions are always present, the other controls are not set to zero but they are considered as constants. So, the following setting is introduced in all the analysis

7 $$\begin{aligned} \begin{array}{l} u_i(t)=u_i^c, \, v_i(t)=v_i^c, \, w_i(t)=w_i^c, \, f_i(t) = f_i^c, \\ z_{i,j}(t)=z_{i,j}^c, \, i,j=1,2,\dots ,N. \end{array} \end{aligned}$$

The resulting study can be also associated to the conditions over a limited time interval in which the controls do not vary sensibly. In addition, as usual for equilibria and stability analysis, the external inputs are set to zero, so that ${\varLambda }_{E_i}(t) = {\varLambda }_{E_i}^c=0$ and ${\varLambda }_{I_{u_i}}(t) = {\varLambda }_{I_{u_i}}^c=0$. Introducing the compact expressions

8 $$\begin{aligned} {{\tilde{c}}}_{i,j} = c_{i,j}(1 - z_{i,j}^c), \, {{\tilde{\mu }}}_i = \mu _i +\sum _{j=1, {j \ne i}}^{N}{{\tilde{c}}}_{i,j}, \, i=1,2,\dots ,N,\nonumber \\ \end{aligned}$$

the system addressed in the present section is

9 $$\begin{aligned} \dot{S_i}= & {} B_i - \beta _i (1-u_i^c) S_iI_{u_i} - {{\tilde{\mu }}}_i S_i +\sum _{j=1, {j \ne i}}^{N}{{\tilde{c}}}_{j,i} S_j, \nonumber \\ \dot{E_i}= & {} \beta _i (1-u_i^c) S_iI_{u_i} - \left( v_i^c + k_i + {{\tilde{\mu }}}_i \right) E_i +\sum _{j=1, {j \ne i}}^{N}{{\tilde{c}}}_{j,i} E_j, \nonumber \\ {\dot{I}}_{u_i}= & {} k_i E_i - \left( v_i^c+h_i \phi _i+\gamma _{I_{u_i}}(1-\phi _i)+{{\tilde{\mu }}}_i\right) I_{u_i} +\sum _{j=1, {j \ne i}}^{N}{{\tilde{c}}}_{j,i} I_{u_j},\nonumber \\ {\dot{I}}_{d_i}= & {} h_i \phi _i I_{u_i}+v_i^c (E_i+I_{u_i})-\left( \gamma _{I_{d_i}}(1+f_i^c) + \mu _{I_{d_i}}(1-w_i^c)\right) I_{d_i}, \nonumber \\ \dot{R_i}= & {} \gamma _{I_{u_i}}(1 - \phi _i) I_{u_i} + \gamma _{I_{d_i}} (1 + f_i^c) I_{d_i} - {{\tilde{\mu }}}_i R_i +\sum _{j=1, {j \ne i}}^{N}{{\tilde{c}}}_{j,i} R_j. \nonumber \\ \end{aligned}$$

### Analysis of the *i*th isolated subsystem

Equilibrium points for system (9), and their stability analysis are here addressed from the point of view of each *i*th submodel, assuming the absence of incoming and outgoing fluxes ($z_{i,j}=1$, $i,j=1,\dots ,N$). Under these positions, the equilibrium points can be computed solving the nonlinear system

10 $$\begin{aligned}&B_i - \beta _i (1-u_i^c) I_{u_i}^e S_i^e - \mu _i S_i^e = 0, \end{aligned}$$

11 $$\begin{aligned}&\beta _i (1-u_i^c) S_i^e I_{u_i}^e - \left( v_i^c + k_i + \mu _i \right) E_i^e = 0, \end{aligned}$$

12 $$\begin{aligned}&k_i E_i^e - \left( v_i^c + h_i \phi _i + \gamma _{I_{u_i}} (1 - \phi _i) + \mu _i \right) I_{u_i}^e = 0, \nonumber \\ \end{aligned}$$

13 $$\begin{aligned}&h_i \phi _i I_{u_i}^e + v_i^c E_i^e + v_i^c I_{u_i}^e \nonumber \\&\quad - \left( \gamma _{I_{d_i}}(1 + f_i^c) + \mu _{I_{d_i}}(1-w_i^c) \right) I_{d_i}^e = 0, \end{aligned}$$

14 $$\begin{aligned}&\gamma _{I_{u_i}}(1 - \phi _i) I_{u_i}^e + \gamma _{I_{d_i}}(1 + f_i^c) I_{d_i}^e - \mu _i R_i^e = 0. \nonumber \\ \end{aligned}$$

From (12) the expression for $E_i^e$,

15 $$\begin{aligned} E_i^e = \frac{\left( v_i^c + h_i \phi _i + \gamma _{I_{u_i}} (1 - \phi _i) + \mu _i \right) }{k_i} I_{u_i}^e, \end{aligned}$$

is obtained and can be used in (11), giving

16 $$\begin{aligned} \begin{array}{l} \beta _i (1-u_i^c) S_i^e I_{u_i}^e \\ - \frac{\left( v_i^c + k_i + \mu _i \right) \left( v_i^c + h_i \phi _i + \gamma _{I_{u_i}} (1 - \phi _i) + \mu _i \right) }{k_i} I_{u_i}^e = 0, \end{array} \end{aligned}$$

from which the two solutions

17 $$\begin{aligned} I_{u_i}^e=0 \end{aligned}$$

and

18 $$\begin{aligned} S_i^e = \frac{\left( v_i^c + k_i + \mu _i \right) \left( v_i^c + h_i \phi _i + \gamma _{I_{u_i}} (1 - \phi _i) + \mu _i \right) }{k_i \beta _i (1-u_i^c)}\nonumber \\ \end{aligned}$$

can be computed. Denoting by the superscript 1 the equilibrium point associated to (17) and by 2 the one associated to (18), using (17) in (10)–(14), the solution

19 $$\begin{aligned} P_i^{e1}=\left( \begin{array}{lllll} \frac{B_i}{\mu _i}&0&0&0&0 \end{array} \right) ^T \end{aligned}$$

is obtained. Equilibrium points like (19), characterized by the absence of any kind of infected people, are usually addressed as *epidemic free*.

The second equilibrium can be obtained starting from (18); setting $S_i^{e2}=S_i^e$, the other components of

20 $$\begin{aligned} P_i^{e2}=\left( \begin{matrix} S_i^{e2}&E_i^{e2}&I_{u_i}^{e2}&I_{d_i}^{e2}&R_i^{e2} \end{matrix} \right) ^T \end{aligned}$$

can be computed as follows

$$\begin{aligned} I_{u_i}^{e2}= & {} \frac{B_i-\mu _i S_i^{e2}}{\beta _i (1-u_i^c) S_i^{e2} }, \\ E_i^{e2}= & {} \frac{B_i-\mu _i S_i^{e2}}{\left( v_i^c + k_i + \mu _i \right) }, \\ I_{d_i}^{e2}= & {} \frac{\left( h_i \phi _i + v_i^c \right) (B_i-\mu _i S_i^{e2})}{\beta _i (1-u_i^c) \left( \gamma _{I_{d_i}}(1 + f_i^c) + \mu _{I_{d_i}}(1-w_i^c) \right) S_i^{e2} } \\&+ \frac{v_i^c (B_i-\mu _i S_i^{e2})}{\left( \gamma _{I_{d_i}}(1 + f_i^c) + \mu _{I_{d_i}}(1-w_i^c) \right) \left( v_i^c + k_i + \mu _i \right) }, \\ R_i^{e2}= & {} \left( \gamma _{I_{u_i}}(1-\phi _i)+\frac{\gamma _{I_{d_i}}(1+f_i^c) \left( h_i \phi _i+v_i^c\right) }{\left( \gamma _{I_{d_i}}(1+f_i^c)+\mu _{I_{d_i}}(1-w_i^c)\right) } \right) \\&\times \frac{B_i-\mu _i S_i^{e2}}{\mu _i \beta _i(1-u_i^c)S_i^{e2} } \\&+\left( \frac{\gamma _{I_{d_i}}(1 + f_i^c) v_i^c (B_i-\mu _i S_i^{e2})}{\mu _i \left( \gamma _{I_{d_i}}(1 + f_i^c) + \mu _{I_{d_i}}(1-w_i^c) \right) \left( v_i^c + k_i + \mu _i \right) } \right) . \end{aligned}$$

An important note is that equilibrium (20) is admissible if and only if all its components are nonnegative. Since for $B_i-\mu _i S_i^{e2} = 0$ the equilibrium (20) coincides with the epidemic-free solution (19), the admissibility of an independent solution is assured only for

21 $$\begin{aligned} B_i-\mu _i S_i^{e2} > 0. \end{aligned}$$

In this case the equilibrium defines a condition in which a certain number of infected individuals is always present and then the epidemic is active. This kind of equilibrium define the so-called *endemic conditions*.

It can be noted that in the epidemic-free condition, the equilibrium point is not dependent on the control actions, since no epidemic is present. On the contrary, the endemic equilibrium point is strongly dependent on the controls, if present.

Once the equilibrium points are known, it is possible to study their stability characteristics. Local conditions can be easily given on the basis of the linear approximation in a neighborhood of each of them.

The first step is the computation of the Jacobian matrix for the system (9) in the isolated conditions considered above, without incoming and outgoing people fluxes. The result is a matrix of the form

22 $$\begin{aligned} J=\left( \begin{array}{c|c} J_{1,1} &{} 0 \\ \hline J_{2,1} &{} J_{2,2} \end{array} \right) , \end{aligned}$$

with

23 $$\begin{aligned} J_{1,1}= & {} \left( {\begin{matrix} -\beta _i(1-u_i^c)I_{u_i}-\mu _i &{} 0 &{} -\beta _i(1-u_i^c)S_i \\ \beta _i(1-u_i^c)I_{u_i} &{} -(v_i^c+k_i+\mu _i) &{} \beta _i(1-u_i^c)S_i \\ 0 &{} k_i &{} -(v_i^c+h_i\phi _i+\gamma _{I_{u_i}}(1-\phi _i)+\mu _i) \end{matrix}} \right) ,\nonumber \\ \end{aligned}$$

24 $$\begin{aligned} J_{2,1}= & {} \left( \begin{matrix} 0 &{} v_i^c &{} v_i^c+ h_i \phi _i \\ 0 &{} 0 &{} \gamma _{I_{u_i}}(1-\phi _i) \end{matrix}\right) , \end{aligned}$$

25 $$\begin{aligned} J_{2,2}= & {} \left( \begin{matrix} - (\gamma _{I_{d_i}}(1 + f_i^c) + \mu _{I_{d_i}} (1-w_i^c) ) &{} 0 \\ \gamma _{I_{d_i}}(1 + f_i^c) &{} -\mu _i \end{matrix}\right) . \end{aligned}$$

Being the eigenvalues of $J_{2,2}$ always real negative ($\lambda _1= - (\gamma _{I_{d_i}} (1 + f_i^c)+ \mu _{I_{d_i}}(1-w_i^c))<0$ and $\lambda _2=-\mu _i<0$), stability of local approximations depends on the different forms assumed by $J_{1,1}$ once computed in $P_i^{e1}$ and $P_i^{e2}$.

Setting, for sake of compactness,

26 $$\begin{aligned} m_i&=(v_i^c+ k_i+ \mu _i), \, \, n_i \nonumber \\&=(v_i^c+ h_i \phi _i + \gamma _{I_{u_i}} (1 - \phi _i) + \mu _i), \end{aligned}$$

the matrix obtained for $P_i^{e1}$ can be written as

27 $$\begin{aligned} J_{1,1}(P^{e1}) = \left( \begin{array}{c|cc} - \mu _i &{} 0 &{} - \beta _i (1-u_i^c) \frac{B_i}{\mu _i} \\ \hline 0 &{} -m_i &{} \beta _i (1-u_i^c) \frac{B_i}{\mu _i} \\ 0 &{} k_i &{} - n_i \end{array} \right) . \end{aligned}$$

along with

From the triangular structure of the matrix and being $- \mu _i<0$, stability is assured once the matrix

28 $$\begin{aligned} \left( \begin{array}{cc} - m_i &{} \beta _i (1-u_i^c) \frac{B_i}{\mu _i} \\ k_i &{} - n_i \end{array} \right) \end{aligned}$$

has eigenvalues, i.e., the roots of the characteristic polynomial equation

$$\begin{aligned} \lambda ^2 + (m_i + n_i ) \lambda + m_i n_i - k_i \beta _i (1-u_i^c) \frac{B_i}{\mu _i}=0, \end{aligned}$$

with negative real part. Thanks to the positiveness of the model parameters and making use of the Descartes’ rule of signs, the required condition holds if and only if

29 $$\begin{aligned} m_i n_i - k_i \beta _i (1-u_i^c) \frac{B_i}{\mu _i}>0. \end{aligned}$$

Easy computations, along with positions in (26), lead to

30 $$\begin{aligned} \frac{B_i}{\mu _i} = S_i^{e1} < \frac{m_i n_i }{k_i \beta _i (1-u_i^c)} = S_i^{e2}. \end{aligned}$$

When the stability of the second equilibrium point $P_i^{e2}$ is investigated, the same procedure as above brings to the computation of the matrix

31 $$\begin{aligned} J_{1,1}=\left( \begin{matrix} - \frac{B_i}{S_i^{e2} } &{} 0 &{} - \beta _i (1-u_i^c) S_i^{e2} \\ \frac{B_i}{S_i^{e2}} -\mu _i &{} -m_i &{} \beta _i (1-u_i^c) S_i^{e2} \\ 0 &{} k_i &{} - n_i \end{matrix} \right) . \end{aligned}$$

Its eigenvalues are given by the roots of the characteristic polynomial

32 $$\begin{aligned} \begin{array}{l} p_{J_{1,1}}(\lambda )= \lambda ^3 + \left( \frac{B_i}{S_i^{e2} } + m_i + n_i \right) \lambda ^2 \\ \qquad + \left( \frac{B_i}{S_i^{e2} } m_i + \frac{B_i}{S_i^{e2} } n_i + m_i n_i - k_i \beta _i (1-u_i^c) S_i^{e2} \right) \lambda \\ \qquad + \frac{B_i}{S_i^{e2} }m_i n_i - \mu _i k_i \beta _i (1-u_i^c) S_i^{e2} \\ \quad = \lambda ^3 + \left( \frac{B_i}{S_i^{e2} } + m_i + n_i \right) \lambda ^2 +\frac{B_i}{S_i^{e2} } (m_i + n_i )\lambda \\ \qquad + \left( \frac{B_i}{S_i^{e2}} - \mu _i \right) m_i n_i. \end{array} \end{aligned}$$

For the Routh–Hurwitz stability criterion, necessary and sufficient conditions to be fulfilled are

33 $$\begin{aligned}&\left( \frac{B_i}{S_i^{e2} } + m_i + n_i \right) \frac{B_i}{S_i^{e2} } (m_i + n_i ) - \left( \frac{B_i}{S_i^{e2} } - \mu _i \right) m_i n_i > 0, \nonumber \\ \end{aligned}$$

34 $$\begin{aligned}&\left( \frac{B_i}{S_i^{e2} } - \mu _i \right) m_i n_i > 0. \end{aligned}$$

It is possible to verify that condition (33) is always satisfied since, after some manipulations, the equivalence with

35 $$\begin{aligned} \frac{B_i}{S_i^{e2} } \left( \frac{B_i}{S_i^{e2} } (m_i + n_i ) + m_i^2 + n_i^2+ m_i n_i \right) + \mu _i m_i n_i > 0 \end{aligned}$$

can be proved. Condition (34) is equivalent to

36 $$\begin{aligned} \frac{B_i}{S_i^{e2} } - \mu _i> 0 \quad \Leftrightarrow \quad \frac{S_i^{e1}}{S_i^{e2}} > 1. \end{aligned}$$

A preliminary observation is that condition (36) is the same as condition (21) and then when the endemic equilibrium exists, it is also locally asymptotically stable. At the same time, being (30) not satisfied, the epidemic-free equilibrium is unstable. The opposite situation arises when (36), and then (21), are not satisfied: the only admissible equilibrium point is the epidemic-free one, and it is locally asymptotically stable.

For the analysis of the epidemic dynamics, it can be useful to derive the model-based expression of the *reproduction number* ${\mathcal {R}}$, which gives the average number of secondary cases produced by a single infected patient in an entire susceptible population [46]. This parameter can be approximatively computed on the basis of the time history of the number of infected individuals to characterize the speed of the epidemic spread [5, 51, 52]: if ${\mathcal {R}}<1$ the epidemic does not spread, reducing autonomously until vanishing; ${\mathcal {R}}>1$ denotes a spreading epidemic with the number of new infected cases growing with a rate related to the magnitude of ${\mathcal {R}}$. An analytical expression, in terms of a model parameters, can be also provided by means of the *next generation matrix* [46]. This procedure is here followed to derive the reproduction number ${\mathcal {R}}^i$ for the isolated *i*-th sub group considered in this Subsection, showing the fulfilment of the above mentioned relationships w.r.t. the stability properties just provided.

According to [46], from system (9) only the equations regarding the infected part of the population $E_i$, $I_{u_i}$ and $I_{d_i}$ are considered; the same assumptions about individual fluxes and controls introduced for the equilibrium and stability analysis are here performed. Denoting by $Z_i(t)=(E_i(t) \quad I_{u_i}(t) \quad I_{d_i}(t))^T$, the following subsystem is defined:

$$\begin{aligned} \dot{Z}_i ={\mathcal {F}}_i-{\mathcal {V}}_i, \end{aligned}$$

where

$$\begin{aligned} {\mathcal {F}}_i=\left( \begin{array}{c} \beta _i(1-u_i^c)S_i(t) I_{u_i}(t) \\ 0 \\ 0 \end{array} \right) \quad \text {and} \quad {\mathcal {V}}_i=\left( \begin{array}{c} {\mathcal {V}}_{i,1} \\ {\mathcal {V}}_{i,2} \\ {\mathcal {V}}_{i,3} \end{array} \right) \end{aligned}$$

with

$$\begin{aligned} {\mathcal {V}}_{i,1}= & {} m_i E_i(t) \\ {\mathcal {V}}_{i,2}= & {} - k_i E_i(t) + n_i I_{u_i}(t) \\ {\mathcal {V}}_{i,3}= & {} - v_i^c E_i(t) - (v_i^c + h_i \phi _i) I_{u_i}(t) + (\gamma _{I_{d_i}}(1 + f_i^c) \\&+ \mu _{I_{d_i}}(1-w_i^c) ) I_{d_i}(t) \end{aligned}$$

Introducing the notation ${\mathcal {F}}^\prime _i=\left( \frac{\partial {\mathcal {F}}_i}{\partial Z_i}\right) _{P_i^{e1}}$ and ${\mathcal {V}}^\prime _i=\left( \frac{\partial {\mathcal {V}}_i}{\partial Z_i}\right) _{P_i^{e1}}$, the reproduction number ${\mathcal {R}}^i$ is given by the eigenvalue with the maximum modulus of the *next generation matrix* ${\mathcal {F}}^\prime _i({\mathcal {V}}^\prime _i)^{-1}$, with

37 $$\begin{aligned} {\mathcal {F}}^\prime _i= & {} \left( \begin{array}{ccc} 0 &{} \beta _i (1-u_i^c) S_i^{e1} &{} 0 \\ 0 &{} 0 &{} 0 \\ 0 &{} 0 &{} 0 \end{array} \right) , \end{aligned}$$

38 $$\begin{aligned} {\mathcal {V}}^\prime _i= & {} \left( {\begin{matrix} m_i &{} 0 &{} 0 \\ -k_i &{} n_i &{} 0 \\ - v_i^c &{} - (v_i^c + h_i \phi _i) &{} (\gamma _{I_{d_i}}(1 + f_i^c) + \mu _{I_{d_i}}(1-w_i^c) ) \end{matrix}} \right) ,\nonumber \\ \end{aligned}$$

39 $$\begin{aligned} ({\mathcal {V}}^\prime _i)^{-1}= & {} \left( \begin{array}{ccc} * &{} 0 &{} 0 \\ \frac{k_i}{m_i n_i} &{} \frac{1}{n_i} &{} 0 \\ * &{} * &{} * \end{array} \right) . \end{aligned}$$

The computation yields

40 $$\begin{aligned} {\mathcal {F}}^\prime _i({\mathcal {V}}^\prime _i)^{-1} = \left( \begin{array}{ccc} \frac{k_i \beta _i (1-u_i^c) S_i^{e1}}{m_i n_i} &{} * &{} 0 \\ 0 &{} 0 &{} 0 \\ 0 &{}0 &{} 0 \\ \end{array} \right) , \end{aligned}$$

in which the greatest eigenvalue can be directly found because of the particular structure. The result is that the basic reproduction number is expressed as

41 $$\begin{aligned} {{{\mathcal {R}}}}^i= \frac{k_i \beta _i (1-u_i^c) S_i^{e1}}{m_i n_i} = \frac{S_i^{e1}}{S_i^{e2}}. \end{aligned}$$

According to its definition, if ${\mathcal {R}}^i>1$ the epidemic spreads within the *i*th population. This corresponds to

42 $$\begin{aligned} {{{\mathcal {R}}}}^i=\frac{S_i^{e1}}{S_i^{e2}} >1, \end{aligned}$$

that is the same condition as (36) providing the stability of the endemic equilibrium (as well as the instability of the disease-free equilibrium). On the other hand, when ${\mathcal {R}}^i<1$ the epidemics reduces and tends to vanish. From (41), this latter condition can be expressed as

43 $$\begin{aligned} {{{\mathcal {R}}}}^i=\frac{S_i^{e1}}{S_i^{e2}} <1 \end{aligned}$$

that guarantees the stability of the disease-free equilibrium too, as required by (30).

### The whole multi-group model

If all the *N* groups are considered together instead of each single one separately as in the previous Subsection 3.1, making reference to system (9), the equilibrium points can be computed solving the *N* systems ($i=1,\dots ,N$)

44 $$\begin{aligned}&B_i - \beta _i(1-u_i^c)S_i^eI_{u_i}^e - {{\tilde{\mu }}}_i S_i^e+\sum _{j=1, {j \ne i}}^{N}{{\tilde{c}}}_{j,i} S_j^e=0, \end{aligned}$$

45 $$\begin{aligned}&\beta _i(1-u_i^c)S_i^e I_{u_i}^e -( v_i^c + k_i + {{\tilde{\mu }}}_i) E_i^e+\sum _{j=1, {j \ne i}}^{N}{{\tilde{c}}}_{j,i} E_j^e=0, \end{aligned}$$

46 $$\begin{aligned}&k_i E_i^e-(v_i^c+h_i \phi _i+\gamma _{I_{u_i}}(1-\phi _i)+{{\tilde{\mu }}}_i)I_{u_i}^e+\sum _{j=1, {j \ne i}}^{N}{{\tilde{c}}}_{j,i} I_{u_j}^e=0, \nonumber \\ \end{aligned}$$

47 $$\begin{aligned}&h_i \phi _i I_{u_i}^e+v_i^c (E_i^e+I_{u_i}^e)-\gamma _{I_{d_i}}(1+f_i^c)I_{d_i}^e-\mu _{I_{d_i}}(1-w_i^c)I_{d_i}^e=0, \nonumber \\ \end{aligned}$$

48 $$\begin{aligned}&\gamma _{I_{u_i}}(1-\phi _i)I_{u_i}^e+\gamma _{I_{d_i}}(1+f_i^c)I_{d_i}^e-{{\tilde{\mu }}}_i R_i^e+\sum _{j=1, {j \ne i}}^{N}{{\tilde{c}}}_{j,i} R_j^e=0. \nonumber \\ \end{aligned}$$

Some conditions on the solutions can be preliminary given by equations inspection. In fact, it is possible to claim

i. for all the admissible solutions, $S_i^e>0$ $\forall i\in [1,N]$. Indeed, supposing $S_i^e=0$ for the generic *i*th system, equation (44) becomes
  49 $$\begin{aligned} B_i + \sum _{j=1, {j \ne i}}^{N} {{\tilde{c}}}_{j,i} S_j^e = 0 \end{aligned}$$
  and, being ${{\tilde{c}}}_{j,i}\ge 0$ and $B_i > 0$, no admissible solutions can be obtained;
ii. if the network is connected, $I_{u_i}^e=0$ for any given $i\in [1,N]$ implies $I_{u_j}^e=0$ $\forall j\in [1,N]$. In other words, any admissible solution for $I_{u}^e$ has either none or all the components equal to zero. In fact, setting $I_{u_i}^e=0$ in (46), one gets
  50 $$\begin{aligned} k_i E_i^e + \sum _{j=1, {j \ne i}}^{N} {{\tilde{c}}}_{j,i} I_{u_j}^e = 0, \end{aligned}$$
  besides $E_i^e = 0$. Being all the coefficients nonnegative, and not all equal to zero from the connected hypothesis on the network, the only admissible solution is
  51 $$\begin{aligned} I_{u_j}^e = 0\qquad \forall j\>:\> {{\tilde{c}}}_{j,i}>0. \end{aligned}$$
  The connection property assures that this result can be propagated through the network, from subsystem *i* to subsystems *j* and so on, giving $I_{u_j}^e = 0, \forall j\in [1,N]$.

Concerning the equilibrium points computation, from (46), for $i=1,\dots ,N$, it is possible to write the compact expression

52 $$\begin{aligned} K E^e - \left( V + {\varPhi } + {\varPsi } + C\right) I_{u}^e = 0 \end{aligned}$$

once the following matrices are introduced

$$\begin{aligned} K= & {} \left( \begin{matrix} k_1 &{} 0 &{} \dots &{} 0 \\ 0 &{} k_2 &{} \dots &{} 0 \\ \vdots &{} \vdots &{} \ddots &{} \vdots \\ 0 &{} 0 &{} \dots &{} k_N \end{matrix} \right) , \, V=\left( \begin{matrix} v_1^c &{} 0 &{} \dots &{} 0 \\ 0 &{} v_2^c &{} \dots &{} 0 \\ \vdots &{} \vdots &{} \ddots &{} \vdots \\ 0 &{} 0 &{} \dots &{} v_N^c \end{matrix} \right) , \\ {\varPhi }= & {} \left( \begin{matrix} h_1 \phi _1 &{} 0 &{} \dots &{} 0 \\ 0 &{} h_2 \phi _2 &{} \dots &{} 0 \\ \vdots &{} \vdots &{} \ddots &{} \vdots \\ 0 &{} 0 &{} \dots &{} h_N \phi _N \end{matrix} \right) \\ C= & {} \left( \begin{matrix} {{\tilde{\mu }}}_1 &{} -{{\tilde{c}}}_{2,1}&{} \dots &{}-{{\tilde{c}}}_{N,1} \\ -{{\tilde{c}}}_{1,2} &{} {{\tilde{\mu }}}_2 &{} \dots &{} -{{\tilde{c}}}_{N,2} \\ \vdots &{} \vdots &{} \ddots &{} \vdots \\ -{{\tilde{c}}}_{1,N}&{} -{{\tilde{c}}}_{2,N}&{}\dots &{} {{\tilde{\mu }}}_N \end{matrix} \right) , \\ {\varPsi }= & {} \left( \begin{matrix} \gamma _{I_{u_1}} (1 - \phi _1) &{} 0 &{} \dots &{} 0 \\ 0 &{} \gamma _{I_{u_2}} (1 - \phi _2) &{} \dots &{} 0 \\ \vdots &{} \vdots &{} \ddots &{} \vdots \\ 0 &{} 0 &{} \dots &{} \gamma _{I_{u_N}} (1 - \phi _N) \end{matrix} \right) . \end{aligned}$$

They are all non-negative diagonal matrices except *C*, which is a positive definite and then non-singular, matrix: due to its structure, it is strictly diagonally dominant (w.r.t. its columns). In fact, from (8) and ${{\tilde{c}}}_{i,j}\ge 0$, one has that $\left| \mu _i+\sum _{j=1, {j \ne i}}^{N} {{\tilde{c}}}_{i,j}\right| \ge$ $\left| \sum _{j=1, {j \ne i}}^{N} {{\tilde{c}}}_{i,j} \right| - \left| \mu _i\right|>$ $\left| \sum _{j=1, {j \ne i}}^{N} {{\tilde{c}}}_{i,j} \right| =$ $\sum _{j=1, {j \ne i}}^{N} \left| {{\tilde{c}}}_{i,j} \right|$.

From (52), the expression

53 $$\begin{aligned} E^e = K^{-1}\left( V + {\varPhi } + {\varPsi } + C\right) I_{u}^e \end{aligned}$$

is obtained. Computing the sum of (44) and (45), for each $i=1,2,\dots ,N$, and collecting them in a vector equation, the resulting expression is given by

54 $$\begin{aligned}&B - C S^e - ( V + K + C) E^e = B - C S^e - (V + K \nonumber \\&\quad + C) K^{-1}\left( V + {\varPhi } + {\varPsi } + C\right) I_{u}^e = 0, \end{aligned}$$

where

55 $$\begin{aligned} B=\left( \begin{matrix} B_1&B_2&\dots&B_N \end{matrix} \right) ^T, \end{aligned}$$

from which we get

56 $$\begin{aligned} S^e&=C^{-1} B - C^{-1} (V + K + C) K^{-1}\left( V + {\varPhi } + {\varPsi } \right. \nonumber \\&\quad \left. + C\right) I_{u}^e. \end{aligned}$$

From Eqs. (45) we get

57 $$\begin{aligned} {\varSigma }(I_u^e) S^e - \left( V + K + C\right) E^e = 0, \end{aligned}$$

where

58 $$\begin{aligned} {\varSigma }(I_u^e)=\left( {\begin{matrix} \beta _1 (1-u_1^c) I_{u_1}^e &{} 0 &{} \dots &{} 0 \\ 0 &{} \beta _2 (1-u_2^c) I_{u_2}^e &{} \dots &{} 0 \\ \vdots &{} \vdots &{} \ddots &{} \vdots \\ 0 &{} 0 &{} \dots &{} \beta _N (1-u_N^c)I_{u_N}^e \end{matrix}} \right) . \end{aligned}$$

Then, exploiting solutions (56) and (53) and introducing the matrix

59 $$\begin{aligned} H=(V + K + C) K^{-1}\left( V + {\varPhi } + {\varPsi } + C\right) , \end{aligned}$$

we get the equation

60 $$\begin{aligned} {\varSigma }(I_u^e) C^{-1} B - {\varSigma }(I_u^e) C^{-1} H I_{u}^e - H I_{u}^e = 0. \end{aligned}$$

Eq. (60) has one solution given by

61 $$\begin{aligned} I_u^e=0, \end{aligned}$$

which substituted in Eqs. (53), (47), (48), (56) provides

62 $$\begin{aligned} E^e= & {} 0, \end{aligned}$$

63 $$\begin{aligned} I_{d}^e= & {} 0, \end{aligned}$$

64 $$\begin{aligned} R^e= & {} 0, \end{aligned}$$

65 $$\begin{aligned} S^e= & {} C^{-1}B, \end{aligned}$$

and then the epidemic-free equilibrium

66 $$\begin{aligned} P^{e1}=\left( \begin{matrix} C^{-1}B&\,&0&\,&0&\,&0&\,&0 \end{matrix} \right) ^T. \end{aligned}$$

Additional equilibrium points can be found computing the non-zero solutions $I_u^e$ of (60) (for which matrix (58) is non-singular) and obtaining all the other components by substituting $I_u^e$ in Eqs. (47), (48), (53), (56). For such solutions, a closed expression is not easy to be found. A numerical approach can be fruitful for each specific case.

The availability of the expression (66) for $P^{e1}$ allows to study its stability characteristics in analytic closed form. Then, the computation of the Jacobian is performed, getting the block-wise form

67 $$\begin{aligned} J(P^{e1})= & {} \left( \begin{array}{c|c|c} -C &{} J_{1,2}(P^{e1}) &{} 0 \\ \hline 0 &{} J_{2,2}(P^{e1}) &{} 0 \\ \hline 0 &{} J_{3,2}(P^{e1}) &{} J_{3,3}(P^{e1}) \end{array} \right) , \end{aligned}$$

where

$$\begin{aligned} J_{1,2}(P^{e1})= & {} \begin{pmatrix} 0&\,&- {\varSigma }(S^{e1}) \end{pmatrix} \\ J_{2,2}(P^{e1})= & {} \begin{pmatrix} -(V+K+C) &{} {\varSigma }(S^{e1}) \\ K &{} -(V+{\varPhi }+{\varPsi }+C) \end{pmatrix} \\ J_{3,2}(P^{e1})= & {} \begin{pmatrix} V &{} (V+{\varPhi }) \\ 0 &{} {\varPsi } \end{pmatrix} \\ J_{3,3}(P^{e1})= & {} \begin{pmatrix} -({\varGamma }+{\varDelta }) &{} 0 \\ {\varGamma } &{} -C \end{pmatrix} \end{aligned}$$

in which

68 $$\begin{aligned} {\varDelta }= & {} \left( \begin{matrix} \mu _{I_{d_1}}(1-w_i^c) &{} 0 &{} \dots &{} 0 \\ 0 &{} \mu _{I_{d_2}}(1-w_2^c) &{} \dots &{} 0 \\ \vdots &{} \vdots &{} \ddots &{} \vdots \\ 0 &{} 0 &{} \dots &{} \mu _{I_{d_N}}(1-w_N^c) \end{matrix} \right) , \end{aligned}$$

69 $$\begin{aligned} {\varGamma }= & {} \left( \begin{matrix} \gamma _{I_{d_1}} &{} 0 &{} \dots &{} 0 \\ 0 &{} \gamma _{I_{d_2}} &{} \dots &{} 0 \\ \vdots &{} \vdots &{} \ddots &{} \vdots \\ 0 &{} 0 &{} \dots &{} \gamma _{I_{d_N}} \end{matrix} \right) , \end{aligned}$$

and

70 $$\begin{aligned} {\varSigma }(S^{e1})=\left( {\begin{matrix} \beta _1 (1-u_1^c) S_1^{e1} &{} 0 &{} \dots &{} 0 \\ 0 &{} \beta _2 (1-u_2^c) S_2^{e1} &{} \dots &{} 0 \\ \vdots &{} \vdots &{} \ddots &{} \vdots \\ 0 &{} 0 &{} \dots &{} \beta _N (1-u_N^c) S_N^{e1} \end{matrix}} \right) . \end{aligned}$$

Thanks to the block structure and the characteristics of *C* and $({\varGamma }+{\varDelta })$, the local stability of $P^{e1}$ is proved once matrix

71 $$\begin{aligned} J_{2,2}(P^{e1})= \left( \begin{array}{cc} -(V+K+C) &{} {\varSigma }(S^{e1}) \\ K &{} -(V+{\varPhi }+{\varPsi }+C) \end{array} \right) \end{aligned}$$

has all its eigenvalues with negative real part. Some considerations about the position of the eigenvalues in the complex plane can be performed making use of the Gershgorin circle theorem on matrix eigenvalues^1^.

In fact, according to the column formulation, it is possible to write

72 $$\begin{aligned} \lambda _i \in {{{\mathcal {D}}}}_i, \qquad i=1,\dots ,N, \end{aligned}$$

where

73 $$\begin{aligned} {{{\mathcal {D}}}}_i=\{z\in {{{\mathcal {C}}}} \>:\> \vert z+ v_i^c+k_i+{{\tilde{\mu }}}_i \vert \le \sum _{j=1, j\ne i}^N {{\tilde{c}}}_{i,j} +k_i \}, \end{aligned}$$

and

74 $$\begin{aligned} \lambda _{N+i} \in {{{\mathcal {B}}}}_{i}, \qquad i=1,\dots ,N, \end{aligned}$$

where

$$\begin{aligned} {{{\mathcal {B}}}}_{i}= & {} \{z\in {{{\mathcal {C}}}} \>:\> \vert z+v_i^c+ h_i\phi _i + \gamma _{I_{u_i}}(1-\phi _i) +{{\tilde{\mu }}}_i \vert \\&\le \sum _{j=1, j\ne i}^N {{\tilde{c}}}_{i,j} +\beta _i (1-u_i^c) S_i^{e1}\}. \end{aligned}$$

Recalling the domains of definition of each parameter, it is possible to give the expressions of the upper bound for the real parts of the eigenvalues. Being the centers real, the minimum $\mathfrak {R}(z)_{min}$ and the maximum $\mathfrak {R}(z)_{Max}$ real parts of possible values of the eigenvalues are the intersections of the circles with the real axis. For the first *N* circles ${{{\mathcal {D}}}}_i$ one has

75 $$\begin{aligned} \mathfrak {R}(z_i)_{min}= & {} -v_i^c-k_i - \mu _i - \sum _{j=1, j\ne i}^N {{\tilde{c}}}_{i,j} \nonumber \\&- \sum _{j=1, j\ne i}^N {{\tilde{c}}}_{i,j} - k_i<0, \nonumber \\ \mathfrak {R}(z_i)_{Max}= & {} -v_i^c-k_i - \mu _i - \sum _{j=1, j\ne i}^N {{\tilde{c}}}_{i,j} \nonumber \\&+ \sum _{j=1, j\ne i}^N {{\tilde{c}}}_{i,j} + k_i = -v_i^c-\mu _i <0, \end{aligned}$$

for $z_i \in {{{\mathcal {D}}}}_i$, $i=1,\dots ,N$, while, for the circles ${{{\mathcal {B}}}}_{i}$ it is possible to write

76 $$\begin{aligned} \mathfrak {R}(z_i)_{min}= & {} - v_i^c - h_i\phi _i - \gamma _{I_{u_i}}(1-\phi _i) - \mu _i - \sum _{j=1, j\ne i}^N {{\tilde{c}}}_{i,j} \nonumber \\&- \sum _{j=1, j\ne i}^N {{\tilde{c}}}_{i,j} -\beta _i (1-u_i^c) S_i^{e1} <0, \nonumber \\ \mathfrak {R}(z_i)_{Max}= & {} - v_i^c - h_i\phi _i - \gamma _{I_{u_i}}(1-\phi _i) - \mu _i \nonumber \\&- \sum _{j=1, j\ne i}^N {{\tilde{c}}}_{i,j} + \sum _{j=1, j\ne i}^N {{\tilde{c}}}_{i,j} + \beta _i (1-u_i^c) S_i^{e1} \nonumber \\= & {} - v_i^c - h_i\phi _i - \gamma _{I_{u_i}}(1-\phi _i) - \mu _i \nonumber \\&+ \beta _i (1-u_i^c) S_i^{e1}, \end{aligned}$$

for $z_i \in {{{\mathcal {B}}}}_i$, $i=1,\dots ,N$. It is possible to conclude that, being the *N* circles ${{{\mathcal {D}}}}_i$ all contained in the real negative part of the complex plane, the epidemic-free equilibrium point is locally asymptotically stable if all the $\mathfrak {R}(z_i)_{Max}$ of (76) are negative, which is true if, $\forall i\in [1,N]$, the following conditions hold

77 $$\begin{aligned} S_i^{e1} < \frac{v_i^c + h_i\phi _i + \gamma _{I_{u_i}}(1-\phi _i) + \mu _i}{\beta _i (1-u_i^c) } = \frac{n_i}{\beta _i (1-u_i^c) }. \end{aligned}$$

Like for each isolated subsystems, it is possible to introduce the reproduction number for the entire network making use of the next generation matrix. The same procedure adopted for the single case is here performed.

According to [46], the same restriction of the full dynamics (9), considered in Subsection 3.1, is taken. It is

78 $$\begin{aligned} \dot{Z} ={\mathcal {F}}-{\mathcal {V}}, \end{aligned}$$

with $$Z=(E\quad I_{u}\quad I_{d})^T$$,

79 $$\begin{aligned} {\mathcal {F}}=\left( \begin{array}{c} \beta _{1}(1-u_1^c)S_1 I_{u_1} \\ \beta _{2}(1-u_2^c)S_2 I_{u_2} \\ \vdots \\ \beta _{N}(1-u_N^c)S_N I_{u_N} \\ \hline 0 \\ \hline 0 \end{array} \right) \end{aligned}$$

and

80 $$\begin{aligned} {\mathcal {V}}= \left( \begin{matrix} {\mathcal {V}}_{1} E \\ -K E + {\mathcal {V}}_{2} I_{u} \\ -V E - (V+ {\varPhi }) I_{u} + ({\varGamma }+ {\varDelta } )I_{d} \\ \end{matrix} \right) . \end{aligned}$$

with

81 $$\begin{aligned} {\mathcal {V}}_{1}=V+ K + C \end{aligned}$$

and

82 $$\begin{aligned} {\mathcal {V}}_{2}=V+ {\varPhi }+ {\varPsi } + C \end{aligned}$$

Then, the derivative of ${\mathcal {F}}$ and of ${\mathcal {V}}$ with respect to the vector *Z* must be computed and evaluated in the disease-free equilibrium:

83 $$\begin{aligned} {\mathcal {F}}^\prime= & {} \left( \frac{\partial {\mathcal {F}}}{\partial Z}\right) _{P^{e1}} = \left( \begin{array}{c|c|c} 0 &{} {\varSigma }(S^{e1}) &{} 0\\ \hline 0 &{} 0 &{} 0 \\ \hline 0 &{} 0 &{} 0 \end{array} \right) , \end{aligned}$$

84 $$\begin{aligned} {\mathcal {V}}^\prime= & {} \left( \frac{\partial {\mathcal {V}}}{\partial Z}\right) _{P^{e1}} = \left( \begin{matrix} {\mathcal {V}}_{1} &{} 0 &{} 0 \\ -K &{} {\mathcal {V}}_{2} &{} 0 \\ -V &{} -(V+{\varPhi }) &{} ({\varGamma }+ {\varDelta }) \end{matrix} \right) , \end{aligned}$$

85 $$\begin{aligned} ({\mathcal {V}}^\prime )^{-1}= & {} \left( \begin{matrix} {\mathcal {V}}_{1}^{-1} &{} 0 &{} 0 \\ {\mathcal {V}}_{2}^{-1} K {\mathcal {V}}_{1}^{-1} &{} {\mathcal {V}}_{2}^{-1} &{} 0 \\ * &{} * &{} ({\varGamma }+{\varDelta })^{-1} \end{matrix} \right) . \end{aligned}$$

The reproduction number ${\mathcal {R}}$ is the eigenvalue with the maximum modulus of

$$\begin{aligned} {\mathcal {F}}^\prime ({\mathcal {V}}^\prime )^{-1} = \left( \begin{matrix} {\varSigma }(S^{e1}){\mathcal {V}}_{2}^{-1} K {\mathcal {V}}_{1}^{-1} &{} {\varSigma }(S^{e1}) {\mathcal {V}}_{2}^{-1} &{} 0 \\ 0 &{} 0 &{} 0 \\ 0 &{}0 &{} 0 \\ \end{matrix} \right) . \end{aligned}$$

Since

86 $$\begin{aligned} \sigma ({\mathcal {F}}^\prime ({\mathcal {V}}^\prime )^{-1})=\sigma ( {\varSigma }(S^{e1}){\mathcal {V}}_{2}^{-1} K {\mathcal {V}}_{1}^{-1}) \cup \{0\}, \end{aligned}$$

the maximum eigenvalue of ${\mathcal {F}}^\prime ({\mathcal {V}}^\prime )^{-1}$ can be found as the maximum eigenvalue of the matrix

87 $$\begin{aligned} {\varUpsilon } = {\varSigma }(S^{e1})(V+{\varPhi }+{\varPsi }+C)^{-1} K (V+K+C)^{-1}. \end{aligned}$$

The analytical computation is not easy and it is preferable to compute ${\mathcal {R}}$ numerically for each specific case. However, for the special case of *N* isolated groups, for which *C* is diagonal, it is possible to simplify ${\varUpsilon }$ so that

88 $$\begin{aligned} {\varUpsilon }= & {} \left( \begin{array}{cccc} \frac{\beta _1 (1-u_1^c) S_1^{e1} k_1 }{m_1 n_1 } &{} 0 &{} \dots &{} 0 \\ 0 &{} \frac{\beta _2 (1-u_2^c) S_2^{e1} k_2}{m_2 n_2 } &{} \dots &{} 0 \\ \vdots &{} \vdots &{} \ddots &{} \vdots \\ 0 &{} 0 &{} \dots &{} \frac{\beta _N (1-u_N^c)S_N^{e1} k_N}{m_N n_N } \end{array} \right) \nonumber \\= & {} \left( \begin{array}{cccc} {{{\mathcal {R}}}}^1 &{} 0 &{} \dots &{} 0 \\ 0 &{} {{{\mathcal {R}}}}^2 &{} \dots &{} 0 \\ \vdots &{} \vdots &{} \ddots &{} \vdots \\ 0 &{} 0 &{} \dots &{} {{{\mathcal {R}}}}^N \end{array} \right) , \end{aligned}$$

yielding

89 $$\begin{aligned} {{{\mathcal {R}}}}=\max _{i\in [1,N]} \left\{ {{{\mathcal {R}}}}^i \right\} . \end{aligned}$$

Eq. (89) expresses the reproduction number of the whole system as the greatest one among all the subgroups of the isolated case. Therefore, the general stability condition (77), in the absence of mobility, implies ${{{\mathcal {R}}}} < 1$ which is the well known condition guaranteeing the local stability of the disease free.

## Numerical analysis of the mobility in Italy from March 9 to October 21, 2020

In this section, the proposed model (2)–(6) is particularized to analyze the COVID-19 evolution in Italy, considering $N=3$ groups (geographical areas) and using real demographic and epidemic data for parameter estimation. Choosing $N=3$ coupled to a summer observation period is motivated to highlight the influence of human movements on the epidemic spread. In the period of interest, which followed the first epidemic wave, a considerable number of movements could be counted across Italy, with non-uniform flow, but with predominant flow orientation from North toward South. We finally note that, in view of the model modularity, the group number *N* could be differently chosen depending on the level of detail required by the analysis. For instance, with the aim of analyzing the epidemic situation in each one of the 20 Italian regions and focusing on the peculiar policies adopted by the local authorities in each area, we could set $N = 20$ specializing the parameters of the submodels on the basis of the local epidemic and social characteristics.

Numerical simulations are performed in order to assess the impact of human mobility and of people transfer on the diffusion of COVID-19 considering three macro-areas of the Italian territory. Each macro-area gathers different Italian regions, as reported by Table 1. Our analysis starts on March 9th 2020, beginning date of the hard lockdown implemented by the Italian government, and covers the summer period with the aim of evaluating the effects of restoring the mobility among regions, evidencing the role played by the holiday exodus and by the reopening of many activities in the late summer in triggering the second pandemic wave.

**Table 1:** Correspondence between macro-areas and Italian regions

Index *i*	Area	Italian regions
1	North	Piemonte, Valle d’Aosta, Liguria, Lombardia, Trentino Alto Adige, Veneto, Friuli Venezia Giulia, Emilia Romagna
2	Center	Toscana, Umbria, Marche, Lazio
3	South	Abruzzo, Molise, Campania, Puglia, Basilicata, Calabria, Sardegna, Sicilia

### Parameter tuning and fitting from data in the interval March 9–June 3, 2020

The time interval selected for parameter fitting from epidemiological data allows to simplify the identification procedure, treating separately the three chosen macro-areas. Indeed, since in the chosen period the mobility was basically forbidden (except for work or necessity reasons) across Italy, we can perform the numerical procedure for the separate identification of the following parameter sets: $\{\beta _i,\phi _i,\mu _{I_{d_i}},\gamma _{I_{u_i}},\gamma _{I_{d_i}}\}$, $i = 1, 2, 3$.

Preliminarily to parameter fitting, the parameters $B_i$, $\mu _i$, $i = 1, 2, 3$, have been evaluated on the basis of the demographic data provided by ISTAT [26]. The per capita death rates $\mu _i$, $i = 1, 2, 3$, have been assumed equal to each other and computed making reference to the mean value, over the period 2011-2018, of the ratio $\mu$ between the number of deaths in Italy in a year and the number of Italians at the end of the same year, i.e., $\mu _1 = \mu _2 = \mu _3 = \mu = 2.81 \cdot 10^{-5}$ days$^{-1}$. Since the Italian population can be considered constant over the relatively “short” period of interest, we assume that birth (plus the net balance between immigration and emigration) approximately compensates for death, and that the average per capita rate of birth and death are comparable, and we compute the net input rate $B_i$ as the product between $\mu$ and the number of individuals $P_i$ in the *i*-th group at a given time (1-1-2020), i.e., $B_i \approx P_i \cdot \mu$, $i = 1, 2, 3$.

Then, according to the sizes (number of persons) of the three populations reported by ISTAT on the 1st of January 2020, $P_1 \approx 27.746 \cdot 10^{6}$, $P_2 \approx 12.016\cdot 10^{6}$, $P_3 \approx 20.597\cdot 10^{6}$, we get $B_1 = 779.67$, $B_2 = 337.65$, $B_3 = 578.79$ (persons $\cdot$ day$^{-1}$).

The parameters $k_i$, $h_i$, $i = 1, 2, 3$, have been fixed on the basis of time constants related to disease progression provided by the World Health Organization and confirmed by the scientific literature [45, 49]. In particular, as previously done in [17], we take $k_i = 1/\tau _{i} = 1/6$ days$^{-1}$ and $h_i = 1/\tau _{s_i} = 1/5$ days$^{-1}$, $i = 1, 2 , 3$, assuming that an exposed individual becomes infective after nearly 3-7 days and that about 5 days are required for the appearance of the first symptoms after the end of the incubation period.

Concerning the relative infectivity of the three populations, the fractions of symptomatic infective people within $I_{u_i}$, $i = 1, 2, 3$, and the per capita rates of death and recovery (for both $I_{u_i}$ and $I_{d_i}$), i.e., the parameters $\beta _i$, $\phi _i$, $\mu _{I_{d_i}}$, $\gamma _{I_{u_i}}$, $\gamma _{I_{d_i}}$, $i = 1, 2, 3$, have been estimated by means of a ordinary least square fitting procedure. For the sake of simplicity, we assumed $\gamma _{I_{u_i}} = \gamma _{I_{d_i}}$ for any *i*. The epidemiological data exploited for the fitting are: i) daily number of diagnosed individuals that are currently positive, ii) total number of recoveries among all diagnosed positives, iii) total number of notified deaths. For each group *i*, such data are respectively reproduced by computing the quantities: a) $I_{d_i}(\delta _{j})$, b) $\int _{\sigma = \delta _{0}}^{\sigma = \delta _{j}} \gamma _{I_{d_i}}(1+f_i(\sigma ))I_{d_i}(\sigma ) d\sigma$, c) $\int _{\sigma = \delta _{0}}^{\sigma = \delta _{j}} \mu _{I_{d_i}}(1-w_i(\sigma ))I_{d_i}(\sigma ) d\sigma$, where $\delta _{0}$ is March 9, beginning date of the national lockdown in Italy, while the j-th notification day $\delta _{j}$ can run until June 3, which is the initial date of restored mobility among regions (about one month after the end of the “strong” lockdown on May 4).

**Fig. 4:**
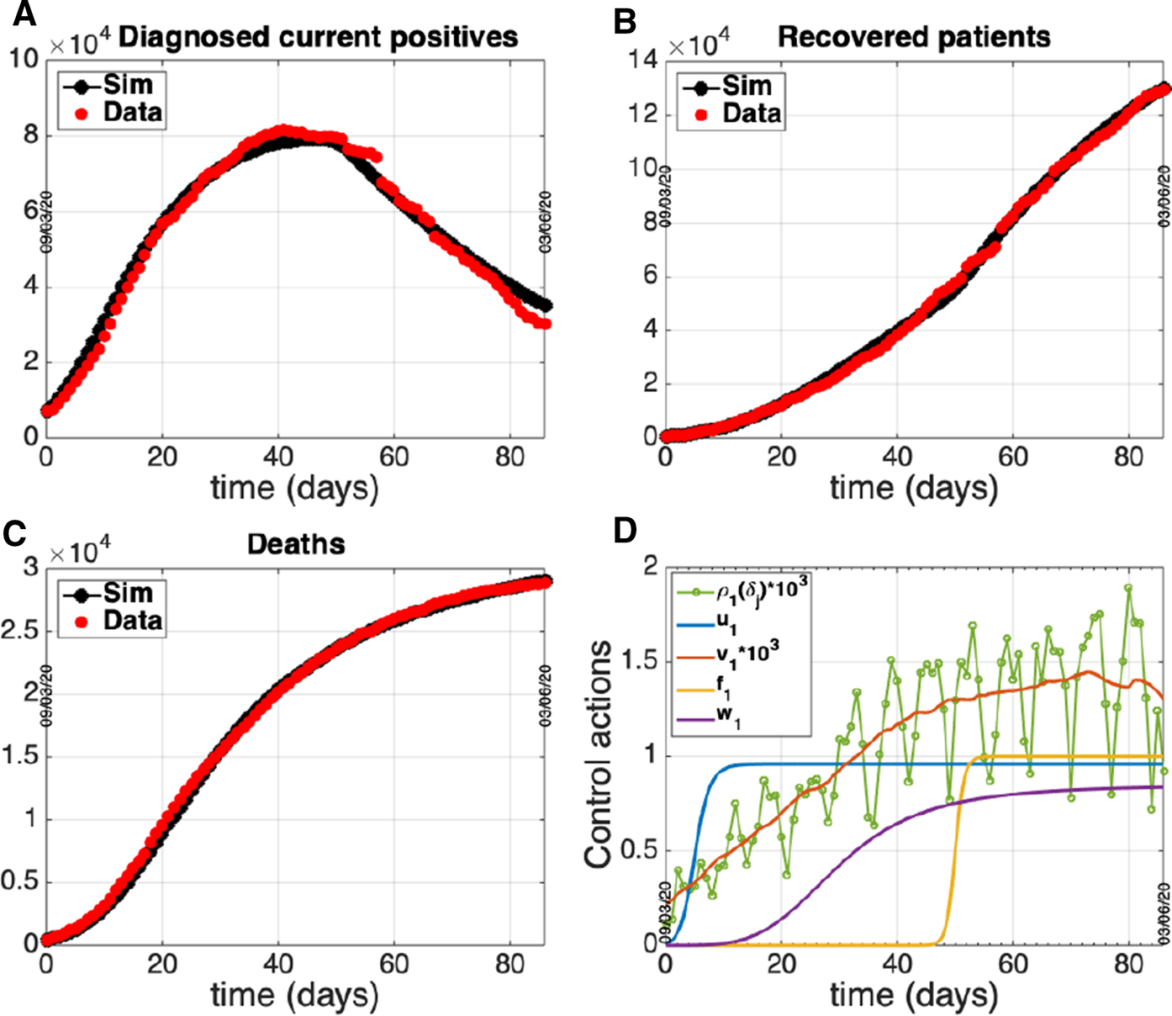
Group 1 - North. Circles: ISS data [18]. Dotted line: model prediction. Panel A: Daily number of diagnosed positives. Panel B: Total number of notified recoveries. Panel C: Total number of notified deaths. Panel D: Time course of the control actions

**Fig. 5:**
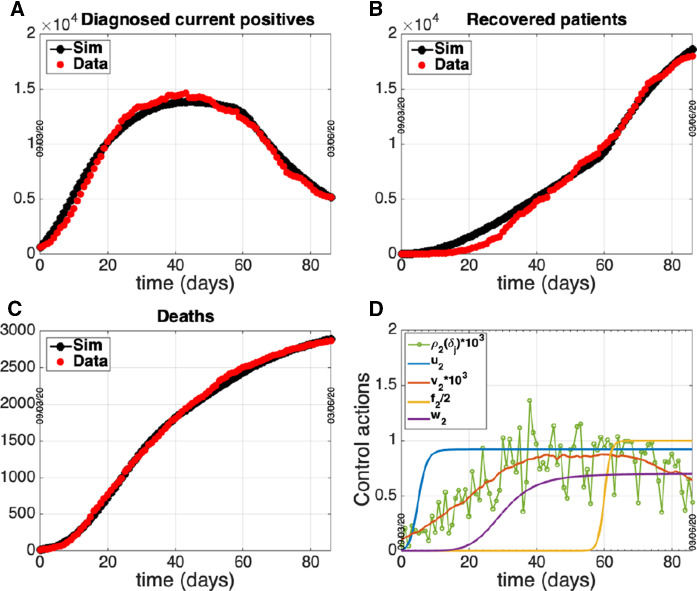
Group 2 - Center. Circles: ISS data [18]. Dotted line: model prediction. Panel A: Daily number of diagnosed positives. Panel B: Total number of notified recoveries. Panel C: Total number of notified deaths. Panel D: Time course of the control actions

**Fig. 6:**
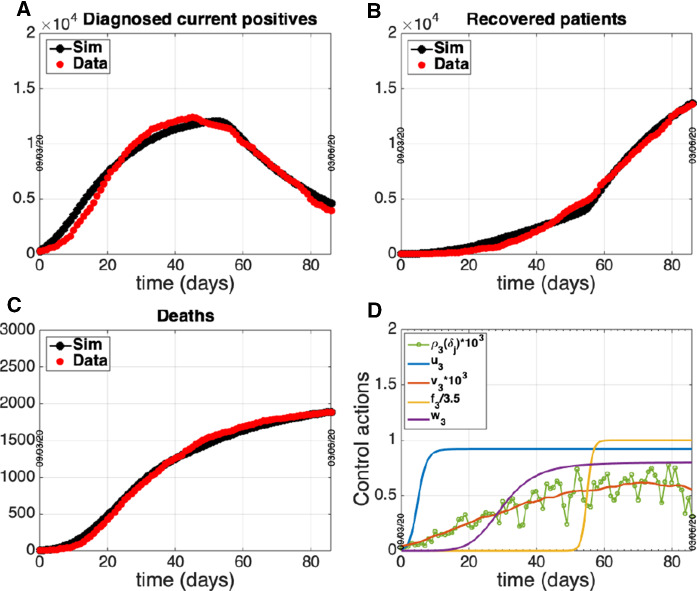
Group 3 - South. Circles: ISS data [18]. Dotted line: model prediction. Panel A: Daily number of diagnosed positives. Panel B: Total number of notified recoveries. Panel C: Total number of notified deaths. Panel D: Time course of the control actions

Suitable time-varying controls, accounting for changes in the social behavior (owing to government restrictions and to increased health risk knowledge), in the swab testing modalities, and in the efficiency of the health system have been exploited for the identification of the parameter sets $\{\beta _i,\phi _i,\mu _{I_{d_i}},\gamma _{I_{u_i}},\gamma _{I_{d_i}}\}$ from data. More in details, the controls $u_i(t)$, $i = 1, 2, 3$, are assumed rapidly increasing as of March 9 as a consequence of the government lockdown decree [17], and reaching the saturation values 0.96, for $u_1$, and 0.92, for $u_2$, $u_3$, about two weeks after the mentioned government act. Such high saturation values are maintained by all the controls $u_i(t)$, $i = 1, 2, 3$, for the entire identification period, so as to represent the achievement of high awareness levels among the Italians at the re-opening. With regard to $v_i(t)$, $i = 1, 2, 3$, these controls are directly inferred from the data. In particular, denoting by $\rho _i(\delta _{j})$ the swab test ratio of group *i* measured at day $\delta _{j}$, it is

90 $$\begin{aligned} \rho _i(\delta _{j}) = \frac{\mathrm{\# \, tests \, at \, time} \, \delta _{j} \, \mathrm{in \, group} \, i }{P_{i}}, \end{aligned}$$

and, for any $t \in [\delta _{j}, \delta _{j+1})$, we take $v_i(t)$ as the linear interpolation between $M_{3}(\rho _i(\delta _{j}))$ and $M_{3}(\rho _i(\delta _{j+1}))$, where $M_{3}(\cdot )$ is the function performing the moving average on a 3-week interval, i.e., a 21-sample moving window, centered in $\delta _{j}$, which allows us to filter out the oscillations in $\rho _i(\delta _{j})$ getting a smoother time course for $v_i(t)$, $i = 1, 2, 3$ (right bottom panels of Figs. 4-6).

Passing to the choice of $w_i(t)$, $f_i(t)$, $i = 1, 2, 3$, a rapid change in their time behavior was required in order to fit adequately the total number of deaths and recoveries of the three groups. The mentioned time variations for $w_i(t)$, $i = 1, 2, 3$, can be explained by assuming an increased medical experience (reached almost at the end of the first wave) in curing the side effects of COVID-19 and in assisting mildly infective people, so avoiding the acute phase of the infection. On the other hand, the change in time of $f_i(t)$, $i = 1, 2, 3$, can be motivated by a likely initial defect in the notification process reporting the daily number of recoveries, especially due to the difficulty of monitoring the infection course of positive people at the beginning of the epidemic in our country.

As far as $z_{i,j}(t)$ is concerned, since uniform mobility restrictions have been implemented in Italy until mid-October, we assume in this analysis $z_{i,j}(t) = z(t)$ for any pair *i*, *j* and for any time. So, the function *z*(*t*) (quantifying the level of mobility between regions) was set to 1.0 throughout the entire estimation interval, since no movement of people was allowed from March 9 to June 3. Obviously, this is a simplifying assumption as some people did actually move for serious or necessity reasons, though observing strict precautionary and distancing measures. However, such a modelling choice allowed to separate the parameter identification of the three ODE submodels, since each dynamical system depends only on the state variables of the related group as long as the condition $z(t) = 1$ is satisfied.

We finally assume a null infective input in each area from abroad during the whole identification period, i.e., ${\varLambda }_{E_i}(t) = {\varLambda }_{I_{u_i}}(t) = 0$, $i = 1, 2, 3$, since strong restrictions and controls for people coming from foreign countries were carried out in Italy during the lockdown.

Concerning the initial conditions of the state variables at $\delta _0$= 9 March 2020, we fixed $I_{d_i}(\delta _0)$ equal to the ISS data on the number of current diagnosed positives of group *i*, while we computed $E_i(\delta _0)$, $I_{u_i}(\delta _0)$, $R_i(\delta _0)$, $i = 1, 2, 3$, according to the ratios $E_i(\delta _0)/I_{d_i}(\delta _0) = I_{u_i}(\delta _0)/I_{d_i}(\delta _0)=11$ and $R_i(\delta _0)/I_{d_i}(\delta _0)=1$, $i = 1, 2, 3$, that we inferred from preliminary simulations on the whole national population [17]. Finally the number of susceptibles of group *i* was computed using the measured $P_i$ by means of the relation $P_i = S_i(\delta _0) + E_i(\delta _0) + I_{u_i}(\delta _0) + I_{d_i}(\delta _0) + R_i(\delta _0)$.

**Table 2:** Estimated model parameters (epidemiological data from March 9 to June 3 [18])

Area	Parameter	Value
North	$\beta _1$	$7.971 \cdot 10^{-9}$ persons$^{-1}$ $\cdot$ day$^{-1}$
	$\phi _1$	0.1145
	$\mu _{I_{d_1}}$	0.0142 day$^{-1}$
	$\gamma _{I_{u_1}}$, $\gamma _{I_{d_1}}$	0.0192 day$^{-1}$
Center	$\beta _2$	$1.794 \cdot 10^{-8}$ persons$^{-1}$ $\cdot$ day$^{-1}$
	$\phi _2$	0.2510
	$\mu _{I_{d_2}}$	0.0067 day$^{-1}$
	$\gamma _{I_{u_2}}$, $\gamma _{I_{d_2}}$	0.0143 day$^{-1}$
South	$\beta _3$	$1.687 \cdot 10^{-8}$ persons$^{-1}$ $\cdot$ day$^{-1}$
	$\phi _3$	0.3176
	$\mu _{I_{d_3}}$	0.0067 day$^{-1}$
	$\gamma _{I_{u_3}}$, $\gamma _{I_{d_3}}$	0.0089 day$^{-1}$

Numerical solutions of the ODE system (2)-(6) were obtained by a MATLAB procedure implementing a classical Runge–Kutta method, exploiting the *ode45* solver. The best fitting procedure is based on *fminsearch* function of MATLAB, suitably modified to guarantee parameter estimates falling in the physical (positive) range. The estimates of the model parameters in each group are reported in Table 2 while the related optimal fitting curves of data i)-iii) are shown in panels A-C of Figs. 4 – 6. The control actions exploited for each fitting are reported in panel D of the same figures.

As a comment to the obtained parameter estimates, we note that the difference of the relative infectivity $\beta _i$ in the three subpopulations is basically attributable to the bilinear form of the incidence rate, $\beta _i S_i I_{u_i}$, and to the different population sizes $P_i$, $i = 1, 2, 3$. Indeed, as the bilinear incidence approximates the standard incidence, $\tilde{\beta _i} S_i I_{u_i}/P_i$, which is normalized to the total population size, the coefficients $\beta _i$, $i = 1, 2, 3$, of our formulation are actually of the order of the inverse of the population sizes $P_i$, $i = 1, 2, 3$ ($\beta _i \sim O(1/P_i)$). So, it is straightforward to verify that the products $\tilde{\beta _i} = \beta _i P_i$, $i = 1, 2, 3$ differ only slightly from each other, ranging about in 0.2-0.3 day$^{-1}$.

Another comment about the estimates of Table 2 concerns the fraction of subjects showing recognizable symptoms within the infective population $I_{u_i}$. It can be noticed that the estimated values of $\phi _i$, $i = 2, 3$ (Center, South) is rather higher than $\phi _1$ (North). This could be explained by the different (smaller) number of swab tests initially implemented in Center-South with respect to the amount of tests performed in North Italy. Indeed, as the virus was prevalent in the Northern area at the beginning of the epidemic outbreak, a very intense test campaign was performed in that area, focusing not only on suspected cases with symptoms (as done in Center-South) but including also the general population even in the absence of recognizable symptoms.

Based on the parameter estimates given in Table 2 and on the expressions of the reproduction number of Sect. 3, we can now provide some predictions on the initial value of such an indicator for the three isolated subsystems and for the interconnected model. As the three areas were actually isolated during the estimation interval ($z_{i,j} = 1$ from 09/03 to 03/06) we can provide an initial estimation of their reproduction numbers, exploiting Eq. (41). In particular, taking into account the control actions on March 9 (first day of lockdown) and the estimated parameters, we obtain ${\mathcal {R}}^1 = 5.45$, ${\mathcal {R}}^2 = 3.49$, ${\mathcal {R}}^3 = 4.94$.

Moreover, we can provide also an evaluation of the basic reproduction number of the whole country by computing the maximal eigenvalue of matrix ${\varUpsilon }$ given by Eq. (87). In particular, setting the onset time of the epidemic spread in Italy at $t_0 <<$ March 9 when the “first” infected appeared in Italy and the mobility was completely active, while no other control actions were implemented ($u^c_i=v^c_i=z^c_{i,j}=0$ in Eq. (87)), we obtain a basic reproduction number ${\mathcal {R}} = \sigma ({\varUpsilon }) = 5.03$. This value is located near the higher literature estimates of the basic reproduction number reported for instance in the study [21].

**Fig. 7:**
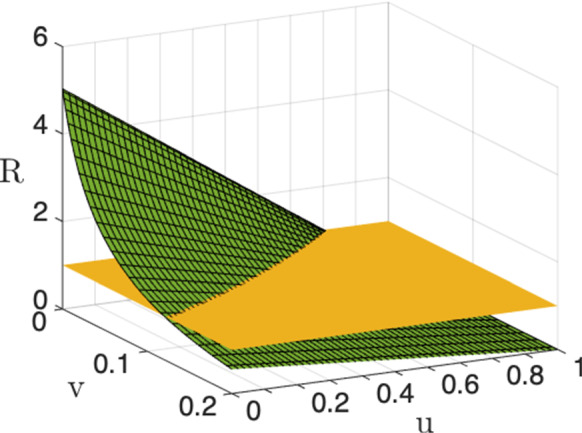
Reproduction number ${\mathcal {R}}$ as a function of $v^c_i=v$ and $u^c_i=u$, $i=1,2,3$ ($z^c_{i,j}=0$, $i,j=1,2,3$)

The identified model parameters, along with the mathematical expression of the reproduction number ${\mathcal {R}}$ given in Sect. 3, allow also to highlight some interesting aspects on the control action intensity which is necessary to contain the disease spread in Italy. Figure 7 shows how the reproduction number of the whole country changes when the intensities of the swab test campaign and of the social contact limitations change. In particular the plot depicts the behavior of ${\mathcal {R}} = \sigma ({\varUpsilon })$ (${\varUpsilon }$ given by Eq. (87)) with respect to $v^c_i=v$ and $u^c_i=u$, $i=1,2,3$, when the mobility is completely active ($z^c_{i,j}=0$, $i,j=1,2,3$). The plot highlights the following aspects:

- in the absence of social contact limitations the containment of the disease spread could be achieved provided that a very intense swab test campaign is performed: indeed, when $u=0$, we get ${\mathcal {R}}<1$ for $v>0.12$; this means that at least the 10$\%$ of the whole population should be tested every day, so requiring about 6 million of swabs per day. This scenario of intense infection tracing is very hard to be actualized, suggesting that some contact limitations are mandatory;
- performing a strong limitation of the social and economic activities which reduces the human contacts more than 80$\%$, could be enough to contain alone the outbreak; in fact, the condition $u > 0.8$ provides ${\mathcal {R}}<1$ independently of the intensity of *v*;
- it is possible to infer a feasible trade-off between social contact limitation and infection tracing, which is not very heavy from both aspects; implementing a reduction of social contacts of 70-80$\%$, i.e., keeping $u \in (0.7, 0.8)$ (note that *u* is at least 0.92 in the lockdown period), allows to contain the outbreak if $v \in (0.0002, 0.02)$. This means that from about 12 thousand (for $u=0.8$) to about 1.2 million (for $u=0.7$) of swabs per day are required.

We notice also that Figure 7 does not significantly change when the mobility level change, even increasing 100 times the coefficients $c_{ij}$ (not shown), suggesting that the stability properties are not sensibly affected by the mobility level. However, as shown by the case study reported in the next section, the human mobility plays an important role in making uniform the spatial distribution of the infection throughout the country, also contributing to a time advance of the second wave.

### Evaluation of the effect of switching on the mobility among areas after June 3rd, 2020

This section is devoted to the numerical simulation of the complete model to investigate the impact that restoring mobility and connections among the groups of regions had on the epidemic evolution of each area. Unrestricted mobility officially started as of June 3 in our country and we aim to reproduce the dynamics of the period following the lockdown phase, quantifying some policies and actions adopted with particular attention to the mobility aspects. Therefore, on the basis of the fitting of the separated models previously obtained, we fix values for the remaining model parameters (representing intergroup exchanges) and we start tuning a tentative mobility framework so as to satisfactorily reproduce the epidemiological data updated with respect to time. Once the model is suitably calibrated with respect to the mobility and control variables, we hypothesize and simulate different evolutionary scenarios that could be envisaged as a consequence of adopting control actions that combine or exclude some restrictions actually adopted in Italy.

In the following, we propose a procedure to derive realistic time courses for the control actions translating into model quantities the social behaviors and sanitary actions. The unknown model variables are adjusted so that the model adequately reproduces the epidemiological data over the chosen period ending on October 21, which is an observation interval that allows to investigate the role played by the increased number of movements in summer on the trigger of the second pandemic wave. Epidemiological data used to adjust the model behavior are the total number of reported positive cases and the daily number of new cases. The reason for considering these data is that they carry “cumulative” information on the total number of subjects notified since the beginning of the observation period and they depend on a reduced number of model parameters. The model quantities representing the mentioned measured data are

91 $$\begin{aligned} C_i(\delta _{j}) = \int _{\delta _{0}}^{\delta _{j}} [h_i \phi _i I_{u_i}(\sigma ) + v_i(\sigma )(E_i(\sigma ) + I_{u_i}(\sigma ))] d\sigma \,, \end{aligned}$$

for the total cases of area *i* at $\delta _j$ and the related increment $C_i(\delta _{j}) - C_i(\delta _{j-1})$ for the daily new cases. Suitable values of $u_i(t)$ and *z*(*t*), as well as of ${\varLambda }_{E_i}(t)$, ${\varLambda }_{I_{u_i}}(t)$, $i = 1, 2, 3$ have been accurately searched by trial and error until satisfactory data reproduction. For simplicity sake, we assume that a single cumulative external flux ${\varLambda }_i(t)$ enters the infected communities and that it is equally split up between $E_i$ and $I_{u_i}$, i.e., ${\varLambda }_{E_i}(t)={\varLambda }_{I_{u_i}}(t)={\varLambda }_i(t)/2$, $i = 1, 2, 3$. Next, ad-hoc time behaviors have to be chosen, according to the available data and official sources, for the coefficients $c_{i,j}(t)$ in order to represent both the “ordinary mobility” (occurring all year round) and the unbalanced transfer of people characterizing the holiday exodus. Concerning the other control actions, $v_i(t)$ is deduced by data on the number of swab tests (as done in the previous section), while $w_i(t)$ and $f_i(t)$ are kept fixed to the last value obtained during the identification period. Note that, as it can be seen from the model equations, the total number of positive cases does not depend on $w_i(t)$ and $f_i(t)$ (as well as on the actual value of the model parameters $\mu _{I_{d_i}}$ and $\gamma _{I_{d_i}}$), so that an accurate tuning of these functions is not required to represent this quantity.

**Fig. 8:**
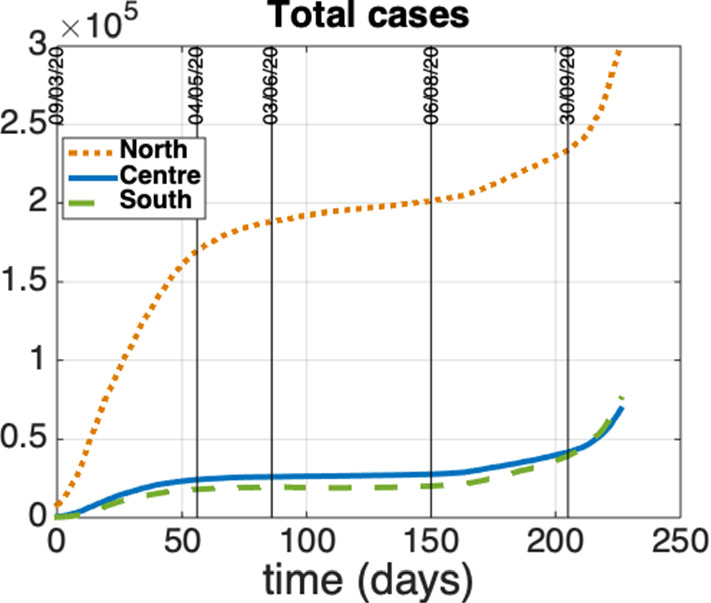
Time course of the total number of cases for the three Italian macro-areas from March 9 to October 21

A first comment that can be made about the total number of positive cases is that their extents in the three geographical areas considered were highly heterogeneous since the beginning of the pandemic outbreak and the same difference was basically maintained until October, as shown in Fig. 8. The total number of cases reported at the beginning of August (around day 150) in the North was about 200000, while a sensible smaller (about one order of magnitude) number of positive cases were reported by Center ($\sim$ 28000) and South (20000).

**Fig. 9:**
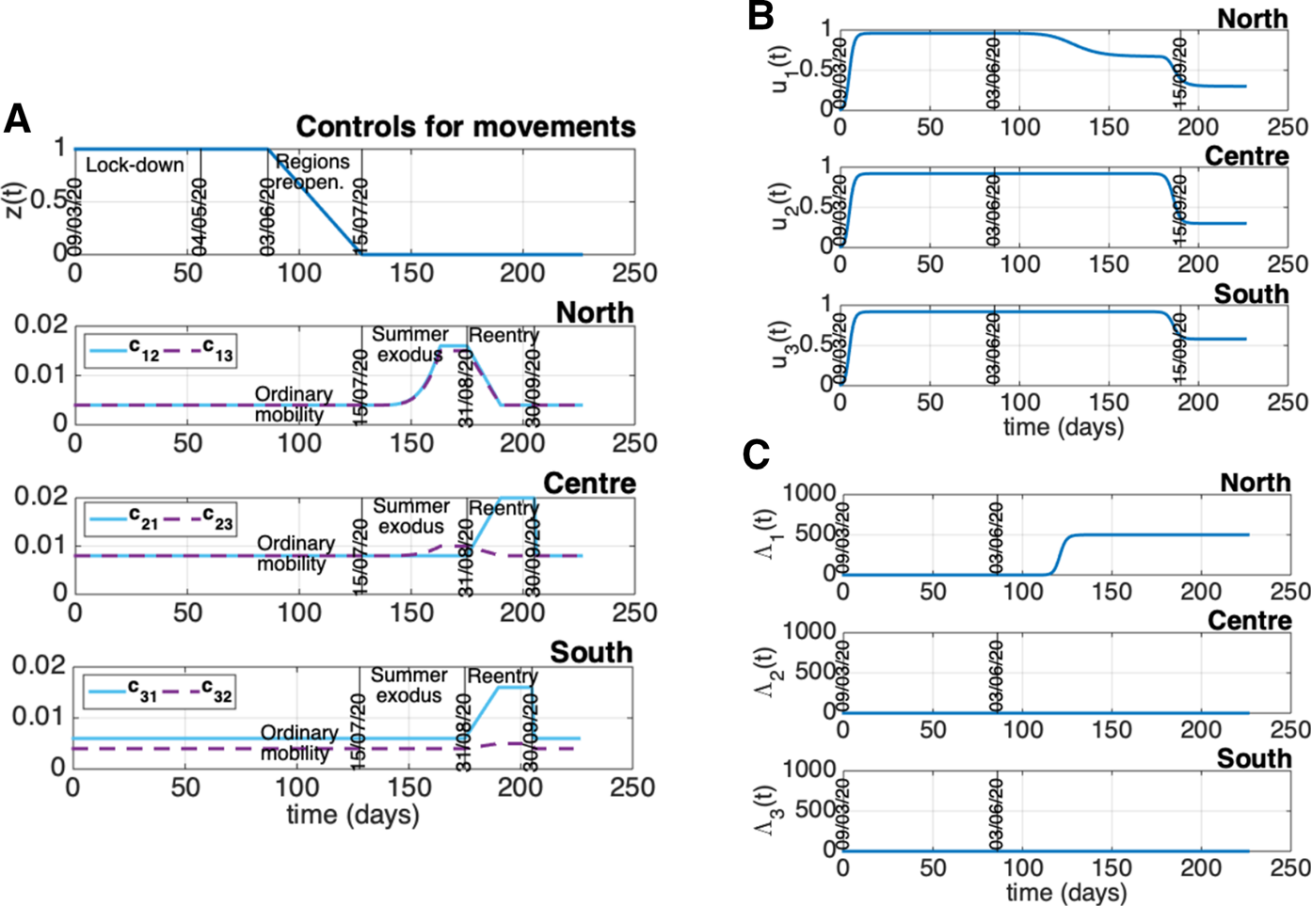
Time course of the control actions adopted to reproduce the data from March 9 to October 21

**Fig. 10:**
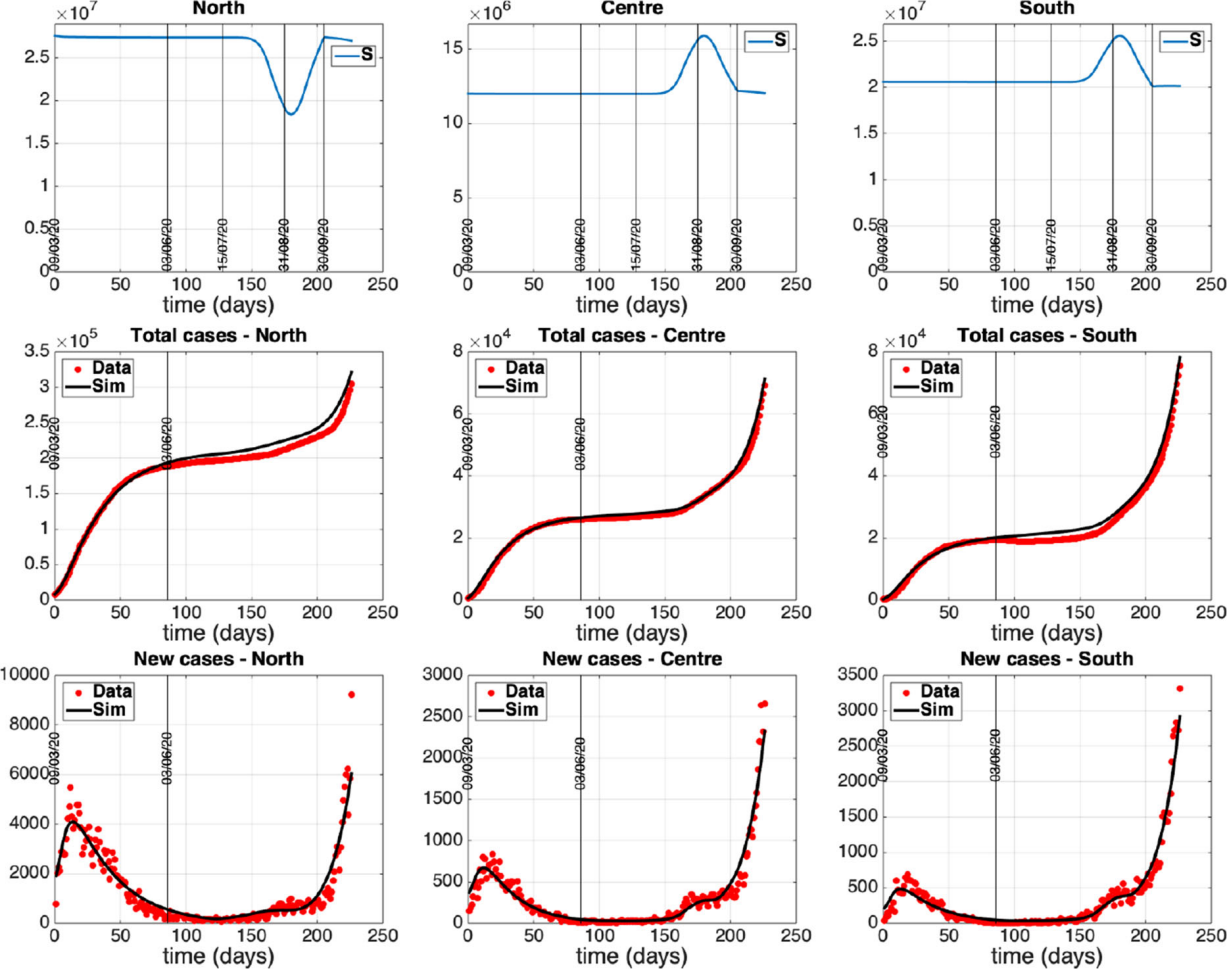
Model reconstruction of the epidemiological data from March 9 to October 21. Upper panels: susceptible population *S*(*t*) northern, central, and southern from left to right. Middle panels: total number of cases. Lower panels: new daily cases. Circles: ISS data [18]. Solid lines: model predictions

**Fig. 11:**
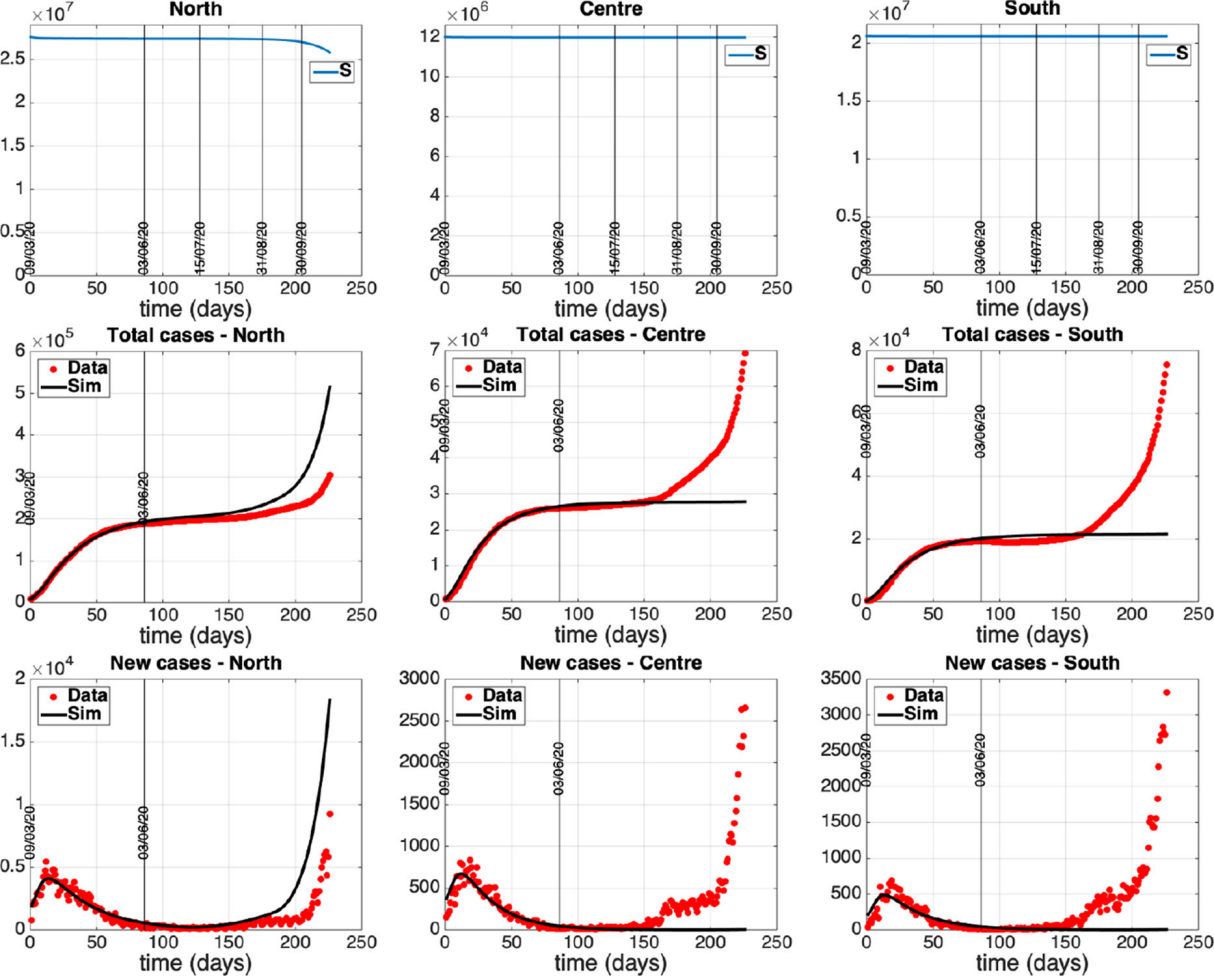
Simulation of scenario (1): $z(t) = 1$ for any *t* in [9 March, 21 October]. The other controls are as in Fig. 9. Upper panels: susceptible population *S*(*t*) northern, central, and southern from left to right. Middle panels: total number of cases. Lower panels: new daily cases. Circles: ISS data [18]. Solid lines: model predictions

**Fig. 12:**
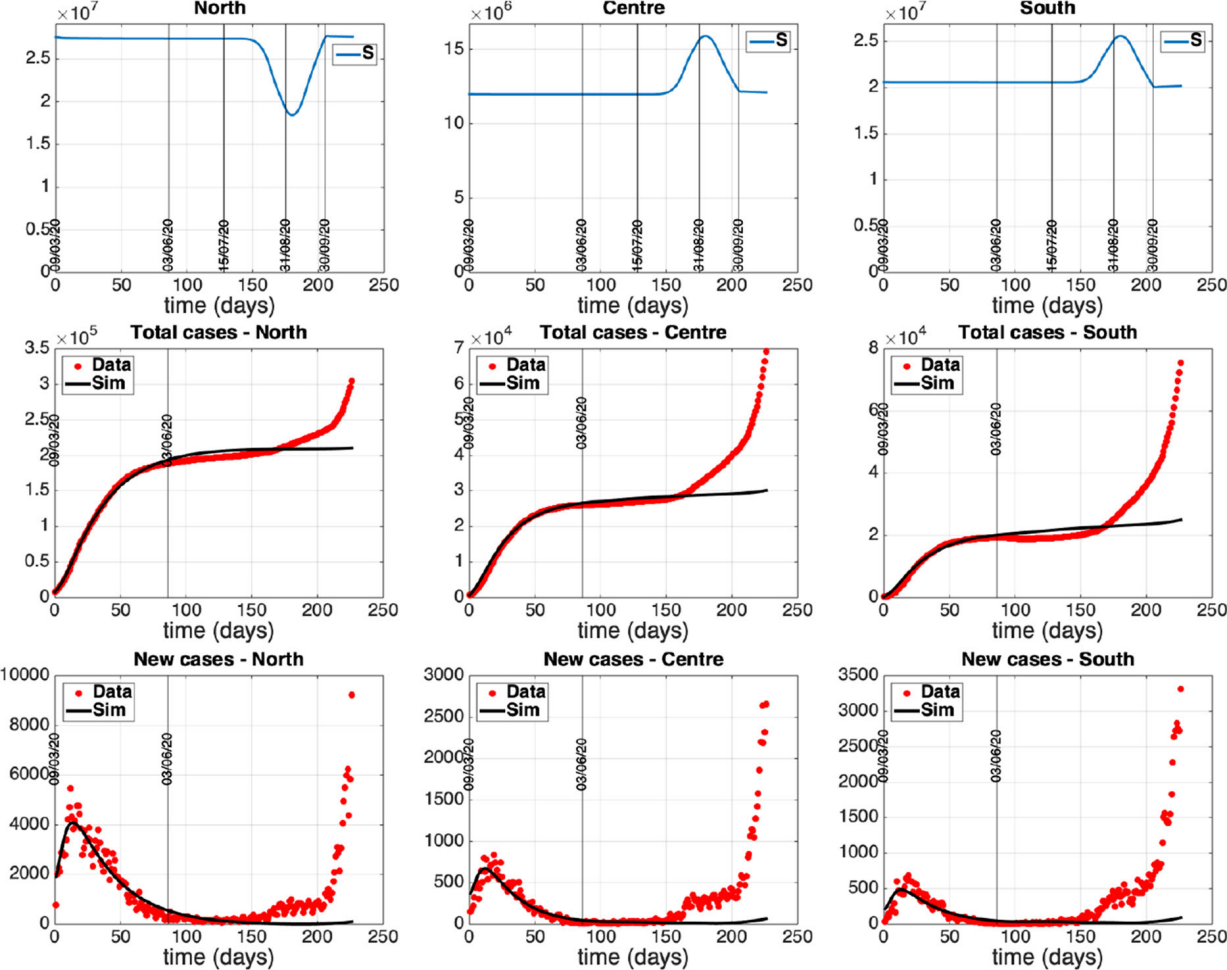
Simulation of scenario (2): ${\varLambda }_{i}(t) = 0$, $i = 1, 2, 3$, for any time and $u_{1}(t) = 0.96$, $u_{i}(t) = 0.92$, $i = 2, 3$, until about September 15. The other controls, as well as $u_{i}(t)$ from the middle of September, are as in Fig. 9. Upper panels: susceptible population *S*(*t*) northern, central, and southern from left to right. Middle panels: total number of cases. Lower panels: new daily cases. Circles: ISS data [18]. Solid lines: model predictions

**Fig. 13:**
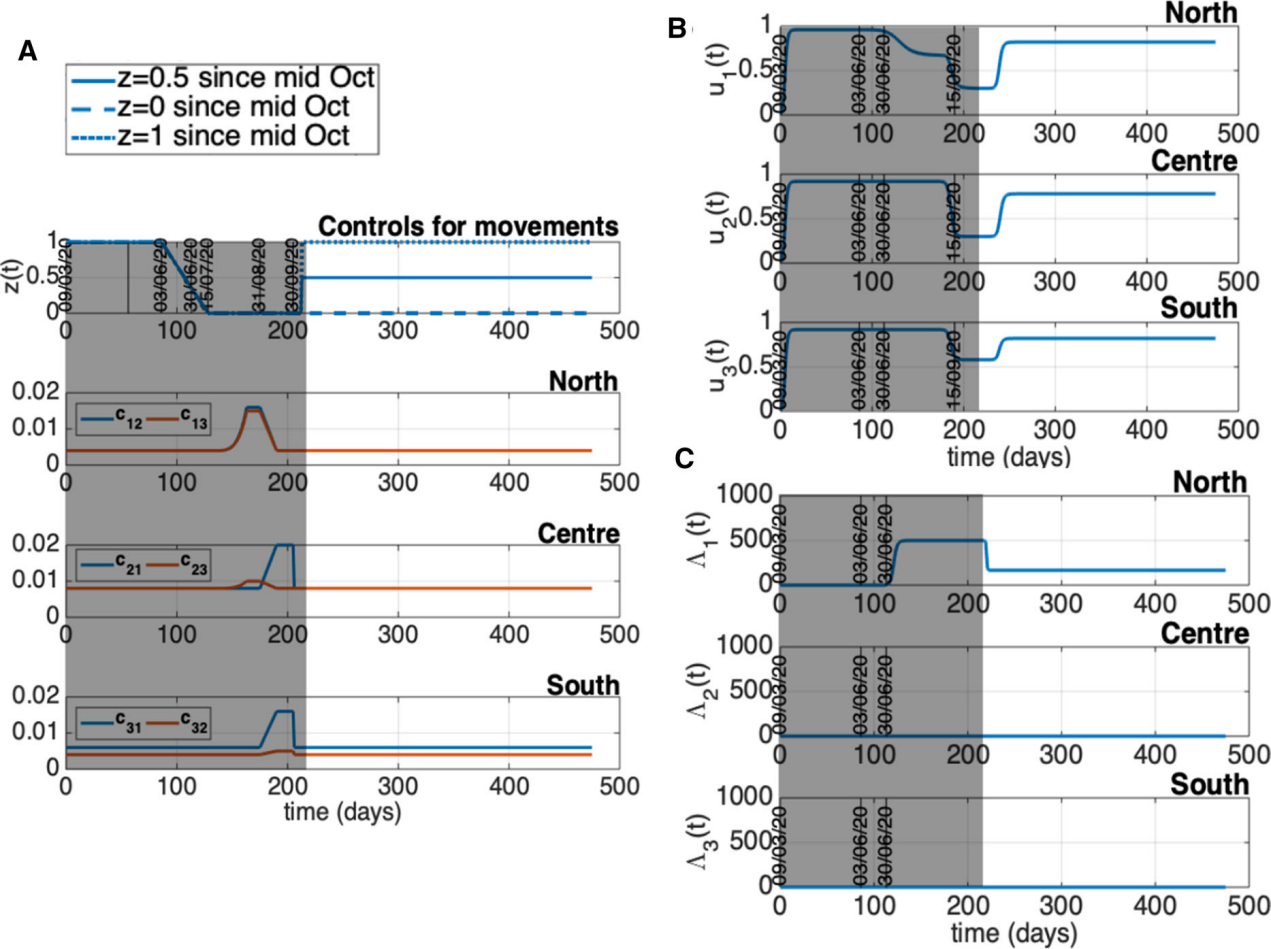
Time behavior of the control actions assumed for the coarse fitting of the epidemiological data until June 2021. $u_i$, $i=1,2,3$, and $i_1$ are changed since mid-October 2020 and different mobility levels are considered from the same date: $z = 0.5$ (reference prediction, solid line) and the extreme conditions $z=0$ (dashed line) and $z=1$ (dotted line)

**Fig. 14:**
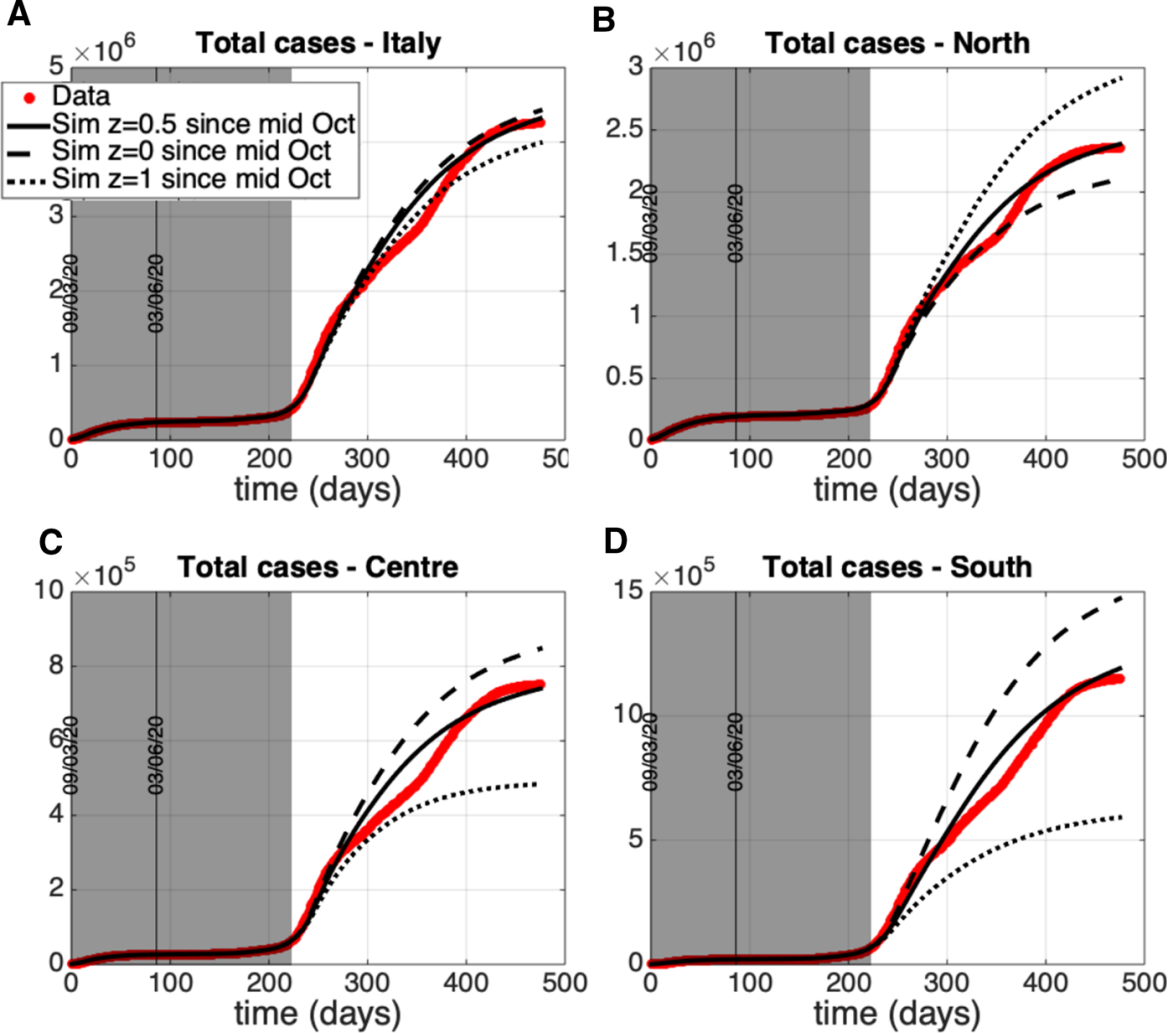
Model prediction on the total number of cases until the end of June 2021. Control actions are changed since mid-October 2020 as reported by Figure 13. Different mobility levels are considered from mid-October 2020: $z = 0.5$ (reference prediction, solid line) and the extreme conditions $z=0$ (dashed line) and $z=1$ (dotted line). Panels: A, whole Italy; B, North; C, Center; D, South. Circles: ISS data [18]. Lines: model predictions

Let us now go into the quantitative details of the control tuning. Figure 9 depicts the time course finally adopted for the control actions, which has been established on the basis of the observations reported in the following. The relatively low number of the daily new cases in the central and southern regions during the mentioned period suggests that neither substantial changes in the social behavior, like the relaxation of precautionary measures or of the social distancing, nor a sensible importation of new infections from outside occurred during the first phase of summer. Therefore, for $i = 2, 3$, the controls $u_i(t)$ should be taken as high as it was during the lockdown, i.e., 0.92, while taking the inputs ${\varLambda }_i(t)$ equal to zero. On the contrary, the number of cases in the North keeps on increasing after the end of the lockdown, although with a slope smaller than before May 4. For this reason, in order to reproduce the data, it is reasonable to hypothesize a decrease of $u_1(t)$ after reopening, or a non-negligible ${\varLambda }_1(t)$, or both these contributions. We assumed both, which means a decrease in $u_1(t)$ (until mid September) and a concomitant increase of ${\varLambda }_1(t)$ just after the reopening. As it can be seen from panels B, C of Fig. 9, the new values set for the inputs ${\varLambda }_i(t)$, $i = 1, 2, 3$, after the 3 June reopening are maintained up to 21 October. Moreover, we assume a sensible change occurring in $u_i(t)$, $i = 1, 2, 3$, (the second reduction for $u_1(t)$) around mid September. On this date the restart of production activities and school re-opening increased the “intra-group” mobility and the consequent social contacts among people. As explained in detail below, this last change of $u_i(t)$, $i = 1, 2, 3$, in addition to the “intergroups” mobility, is actually a necessary assumption in the simulation to explain the sharp increment of cases starting from the end of September in all the three areas (see Fig. 8) .

As far as the mobility controls *z*(*t*) and $c_{i,j}(t)$, $i \ne j$, $i, j = 1, 2, 3$ are concerned, they are chosen on the basis of the following considerations. First, according to the government decrees allowing people to travel as of June 3, we assume that the mobility is restored gradually along about forty days, so that *z*(*t*) starts decreasing from 1 (no mobility) on June 3 going to 0 (complete mobility) on July 15. In addition, we need to quantify the “regular mobility”, i.e., the amount of people transfers for study and work, or just for visiting under “ordinary” conditions. So, in order to model the “regular mobility” we keep the coefficients $c_{i,j}(t)$, $i \ne j$, $i, j = 1, 2, 3$, constant, say $c_{i,j}(t)={\bar{c}}_{i,j}$, $i \ne j$, $i, j = 1, 2, 3$, before and after the holidays (which means out of the interval 15 July–30 September). Such constant coefficients are tuned in order to provide approximately 100000 persons travelling each day and between each pair of areas, paying attention in balancing the macro-areas demographies, i.e., assuming a constant number of people in each of them. In particular, we choose ${\bar{c}}_{1,2} = 4\cdot 10^{-3}$, ${\bar{c}}_{1,3} = 4\cdot 10^{-3}$, ${\bar{c}}_{2,1} = 8\cdot 10^{-3}$, ${\bar{c}}_{2,3} = 8\cdot 10^{-3}$, ${\bar{c}}_{3,1} = 6\cdot 10^{-3}$, ${\bar{c}}_{3,2} = 4\cdot 10^{-3}$ (see Fig. 9, panel A).

From the beginning of August till the end of September, a remarkable mass departure of Italians from North to Center and South took place, with a sensible delay with respect to previous years and with a remarkable increment of internal travelling toward the Southern seas or mountains. Indeed, a statistical analysis reported in September 2020 by ENIT (the Italian National Agency for Tourism) shows that almost 24 millions of Italian went to Center-South for their holidays [19]. For this reason, as of July 20 we impose changes to the coefficients $c_{i,j}(t)$, $i \ne j$, $i, j = 1, 2, 3$, to reproduce an unbalanced demographic movement toward the Central and Southern populations whose size increases of some millions of persons (the northern population was reduced accordingly). Conversely, from about August 31 the mobility across Italy progressively changed again up to mid September, producing the opposite migration of people from Center-South to North. The summer exodus is thought to terminate at the end of September. A temporal scheme of the model quantities so assumed is illustrated in Fig. 9, while their simulated effect on the susceptible populations (that basically coincide with the entire resident populations of the areas) is shown by the upper panels of Fig. 10.

The time course of the control actions assumed as described (see also Fig. 9) allows to reproduce well the data behavior by means of the model. This is shown in Fig. 10 for the total number of cases (middle panels) and for the daily number of new cases (lower panels) reported from June 3 to October 21. The main comment that we can give is that the combination of the summer exodus from North to Center-South along with the actually enhanced virus circulation in the North ($u_1(t)$ lowered and ${\varLambda }_1(t)$ increased in the model) produces the joint spread and increment of positive cases throughout the three areas, and especially in Center-South. Indeed, in the interval 150-200 days the total cases present a net slope change (Fig. 10, middle panels) while the daily number of new cases take a more bell-shaped course (lower panels of Fig. 10). Overall, such increment of positive cases was rather limited, which in our opinion proves that the general mobility increment during the summer holidays was not sufficient to directly induce the feared second wave. However, they did probably contribute to the second wave since the fast increase of cases after September 15 can be actually reproduced only by combining the further sensible reduction of containment measures (i.e., $u_i(t)$) after that date with the assumptions on mobility and social behavior made for the summertime. This combination of effects can be demonstrated also by the predictions reported below, which we construct by simulating the removal of one control variable at a time.

So, let us now evaluate the impact of the single control actions, evidencing how the combination of the different controls assumed above is necessary to obtain a sufficiently accurate reconstruction of the data. In particular, our aim is to evaluate how different the situation could have been by adopting different control strategies. The following two scenarios are schematized by model simulation: (1) epidemic evolution keeping the restricted inter-regional mobility even after June 3; (2) epidemic evolution with restored mobility, but supposing that stronger precautionary measures and restrictions on social contacts are applied. Scenario (1) can be simulated setting $z(t) = 1$ for any time (until October 21). Scenario (2) can be modelled by fixing ${\varLambda }_1(t) = 0$ for any time and keeping $u_1(t) = 0.96$ at least until the middle of September (instead of allowing $u_1(t)$ to go down to 0.67 after the reopening, and before mid September, as in the reference simulation). Figures 11 and 12 report the model predictions obtained for the two scenarios (1) and (2) supposing valid the variations mentioned above w.r.t. the reference situation of Fig. 10. From the results of Fig. 11 it is evident that, if the inter-regional mobility had been prevented (i.e., keeping closed the borders of the three areas), a second wave would have occurred sooner in the North, where the virus circulation was higher than elsewhere, but it would have spared the other areas from a sharp increment of infections (at least until October 21). So the simulation evidences the role of the holiday mobility in the diffusion of the epidemic along the Italian territory, making the number of infections more uniformly distributed in the country and delaying the second wave. Conversely, from Fig. 12 we can conclude that, if stricter rules for the social contacts and stronger precautionary measures had been imposed at the reopening (after June 3), the second wave would have been avoided in all the three groups until October 21. Note that the reduction of the controls $u_i(t)$, $i = 1, 2, 3$, after the middle of September is not sufficient to produce the rapid increase that we actually observe in the real data over a month (from 15/09 to 21/10). Indeed, from the middle and lower panels of Fig. 12 we can notice a barely perceptible increase of the simulated cases (both total and new daily cases) at the end of the considered time interval (indeed, the same number of cases are basically obtained until October 21 without reducing $u_i(t)$ from the middle of September). This means that the second wave is slowly starting from the final days of the considered time interval, because of the reduction of $u_i(t)$, $i = 1, 2, 3$, at the middle of September, but if this had been the case the disease progression could have been slower giving more time to the government for effective interventions to limit the impact of the second wave.

## Numerical analysis of the mobility impact after October 21, 2020

The model presented in this paper has been used to evaluate the contribution that the summer holidays, with their largely incremented mobility, had on the virus spread along Italy until mid October 2020 (i.e., until the second wave onset). In the present section, the model capability to capture and describe the mobility even in the subsequent time up to nowadays is discussed. Maintaining the aggregation of Italian regions into three groups when a color based mobility characterization of each region is actually adopted since November 2020 clearly brings to averaged results. It is worthy to note that the model (2)–(6) could be specialized to describe the twenty regions division of Italy once a new identification procedure is performed. Keeping the model as used in Sect. 4, with the parameter values as estimated in Sect. 4.1, new predictions on the human mobility after summer 2020 are presented once a coarse tuning of the control actions is performed accounting for new social, economic and mobility restrictions introduced in Italy to mitigate the incoming second wave. To this aim, starting from the last values of the controls reported in Figure 9 we impose the following variations at mid-October: (i) an increase of the social contact limitations $u_i$, $i=1,2,3$, up to 0.8; (ii) a reduction of $i_1$ to one third of the maximal value reached after the first reopening; (iii) a reduction of 50$\%$ of the mobility level ($z=0.5$). The variations imposed to the mentioned controls are kept constant until the end of June 2021; moreover, the values of the actions $v_i$, $i=1,2,3$, are driven by the data on the swab tests administered in the three macro-areas, as done before in Sect. 4. A compromise between a fine tuning of the control actions and the averaged description over the aggregated three macro-areas is adopted. The assumed controls and the prediction results are shown in Figures 13 and, respectively, Fig. 14 (solid lines); such a coarse control tuning provides a satisfactory model prediction which roughly reproduces the total number of cases (see the solid lines of Figure 14, related to $z=0.5$, compared to the real data). Starting from this reference prediction, we evaluate how much the total number of cases could have been different in each area by changing the mobility level between the two extreme conditions $z=0$ and $z=1$. Indeed, Figure 14 confirms that the total number of infected patients could have been sensibly different in the whole country and in each area at the end of June 2021, depending on the level of human transfers among the three groups. Indeed, passing from $z=0$ (dashed line, free mobility) to $z=1$ (dotted line, mobility forbidden), we obtain a reduction of about $-10\%$ of the total cases at the end of June 2021 in the whole Italy and, in particular, the following local variations of cases in the three areas: $+38.5\%$ in the North, $-42.9\%$ in the Center and $-59.8\%$ in the South. The strongest impact of the mobility between the three areas is obtained in the South, where the level of local infected people was lower than those of the other areas at mid October (compare the number of cases w.r.t. the local population size) and a significant import of infected could change the time evolution of cases in this area. Moreover, the variation of cases (which are given by the integral of the incidence) evaluated for the whole country suggests that the mobility has also a sort of multiplying effect on the contagion in addition to the spatial infection distribution.

As a final and general remark about the application of the model presented here, it is worth stressing the interest of the proposed COVID-19 analysis not only to explain the current emergency, but also to understand the underlying dynamics, in view of possible similar future situations. As noted by important theoretical studies such as [1, 22, 32, 36, 50], the evolution of any pandemic (and particularly the current one) strongly depends on the population actions in the early phase; nevertheless, the model provides a quantitative tool for evaluating the impact of the applied containment measures, in particular of the mobility restrictions, in different epidemic scenarios.

## Concluding remarks

In this paper, an enriched version of the classical SEIR model, including the effects of asymptomatic individuals on the COVID-19 outbreak, is proposed to describe the spread of a virus among different geographically defined populations, with the aim of analyzing the consequences of inter-regional people mobility on the virus transmission.

After an in-depth model analysis, we present a case study, which is interesting by itself and also as a possible future scenario, considering the effects of the people fluxes among Italian macroareas in summer 2020. During that period, the mobility restrictions were relaxed and a considerable number of movements with predominant flow orientation from North toward Center-South (in early summer, and backward, in late summer) occurred.

By using official Italian epidemiological data, it is evidenced the capability of the proposed modelling of capturing the effects of individual mobility among regions characterized by different local evolutions and effects of government decrees to regulate and limit such inter-regional mobility.

The study of the expression of the reproduction number showed its dependency from the model parameters; in particular, it is stressed the influence of the swab test campaign and its relation with the changes in social contacts limitations, showing the possibility of an acceptable trade-off between social contacts limitation and infection tracing once an high number of swab test is processed.

The numerical results obtained by *ad hoc* model simulations evidenced the capability of capturing the effects of the individual mobility among regions, characterized by different local evolutions, and the consequences of government decrees to regulate and limit such inter-regional mobility. It is proved that the mobility increment during summer 2020 was not sufficient to immediately induce the second wave, but it was probably decisive to its onset when the mass departure effect was combined with the restart of the productive activities and school reopening. Preventing the mobility among the macro-areas considered would have probably saved the Center-South from a sharp contagion increase after the summer, while producing a time advance of the second wave in the North. Prolonging the analysis along the next interval (mid-October 2020–end of June 2021) we evidenced the role of the human mobility in multiplying the number of infections in the whole country.

Future developments of the present study in progress are represented by the use of the identified models and the quantified contribution of mobility, social and economic activities, and schools, to analyze and predict the effects of the combination of containment actions in different possible scenarios. The possibility of a deeper description increasing the resolution adopting the twenty regions division is also under investigation, to evaluate the effects of the short–medium distance mobility too.

## Data Availability

Data available in public repositories cited in the references.

## Footnotes

1 The eigenvalues of a $n \times n$ matrix A with entries $a_{i,j}$ belong to at least one of the *n* discs ${{{\mathcal {D}}}}_i$, $i=1,\dots ,n$, where ${{{\mathcal {D}}}}_i=\{z\in {{{\mathcal {C}}}} \>:\> \vert z-a_{i,i}\vert \le \sum _{j=1, j\ne i}^n \vert a_{i,j}\vert \}$ for a row formulation, or ${{{\mathcal {D}}}}_i=\{z\in {{{\mathcal {C}}}} \>:\> \vert z-a_{i,i}\vert \le \sum _{j=1, j\ne i}^n \vert a_{j,i}\vert \}$ for a column based version.
